# Supplementary material for: Mouse Spermatogenic Stem Cells Continually Interconvert between Equipotent Singly Isolated and Syncytial States
Source: Cell Stem Cell. 2014 May 1;14(5):658–72. doi: 10.1016/j.stem.2014.01.019 (PMC4010676; doi:10.1016/j.stem.2014.01.019)
Supplement: Document S1. Supplemental Experimental Procedures and Figures S1–S5 [file mmc1.pdf]

Cell Stem Cell, Volume *14*

Supplemental Information

Mouse Spermatogenic Stem Cells Continually Interconvert between Equipotent Singly Isolated and Syncytial States

Kenshiro Hara, Toshinori Nakagawa, Hideki Enomoto, Mikiko Suzuki, Masayuki Yamamoto, Benjamin D. Simons, and Shosei Yoshida

1. Supplemental Figures and Legends

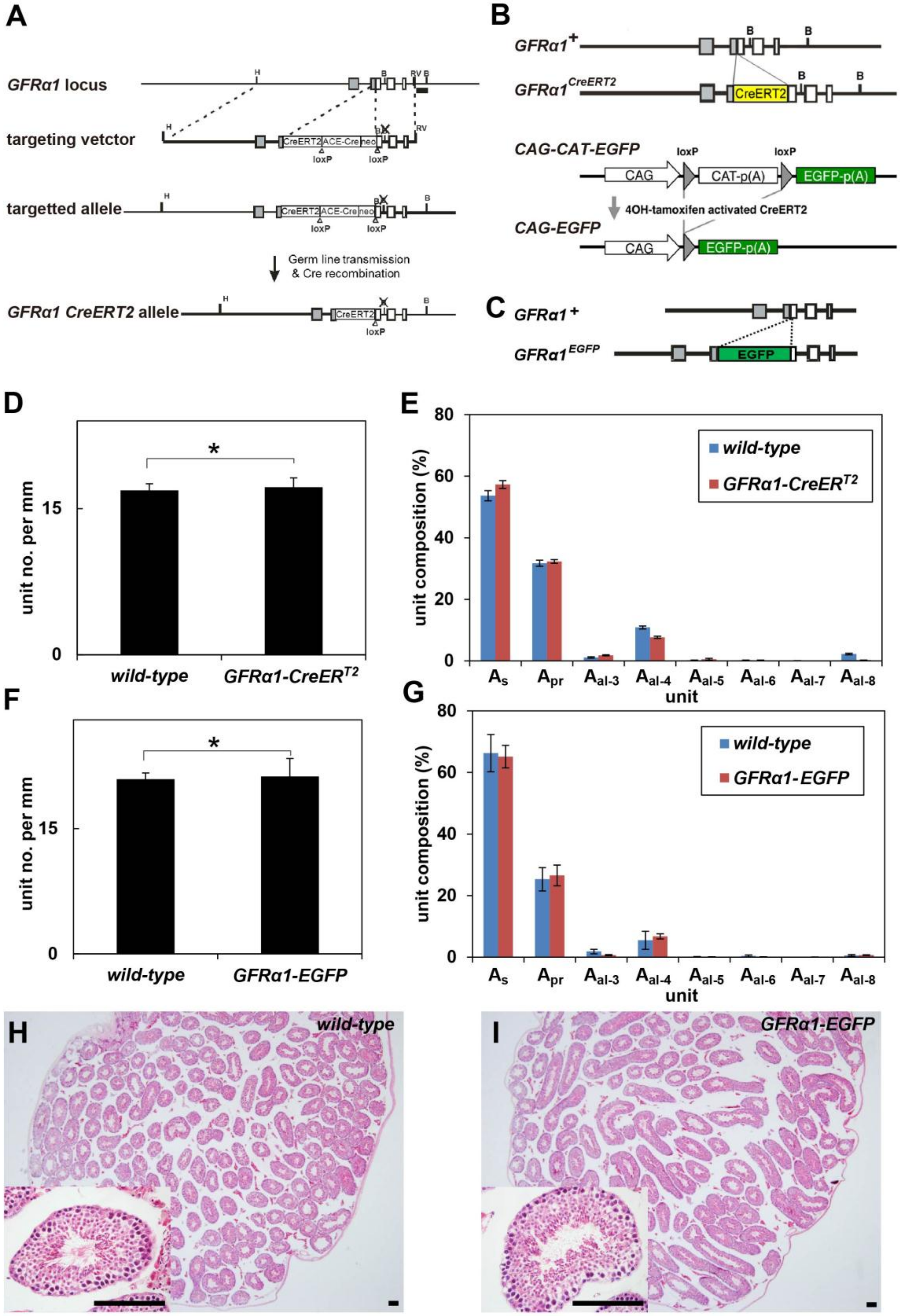

Fig. S1

Fig. S1: Supplemental data for the animals used in this study, related to Figs. 2, 3, 5, 6, and Experimental Procedures.

(A) Generation of *GFRα1-CreER*^{T2} Knock-in allele. A gene cassette composed of *CreER*^{T2} *cDNA-BGH poly(A)* followed by a floxed *angiotensin converting enzyme (ACE)-Cre* and *Tn5-neo* cassette were introduced into the second exon of the *GFRα1* gene by gene targeting as described previously (Enomoto et al., 2004). This insertion deleted 95 nucleotides containing the 5'-UTR, the initiator Met and the signal sequences. Upon germ line transmission of the targeted allele, floxed *ACE-Cre* and *Tn5-neo* were self-excised by ACE-Cre-mediated recombination in ES-cell-derived sperm (Bunting et al., 1999), culminating in the generation of *GFRα1-CreER*^{T2} allele. All animal procedures regarding the generation and characterization of *GFRα1-CreER*^{T2} allele were conducted with approval of the Washington University Animal Studies Committee and Animal Research Committee of the RIKEN Center for Developmental Biology. **(B)** Structure of *GFRα1* alleles and *CAG-CAT-EGFP* transgene. Injection of 4OH-tamoxifen into *GFRα1-CreER*^{T2}*; CAG-CAT-EGFP* mice induces temporal Cre activity in GFRα1+ cells, which causes permanent expression of *GFP* under *CAG* promoter, following excision of *CAT* gene floxed by the loxP sequences. **(C)** Structure of the *GFRα1-EGFP* knock-in allele carried heterozygously by the mice used for live-imaging (Uesaka et al., 2007). **(D-G)** No obvious effect of heterozygousity in *GFRα1* locus on the average GFRα1+ unit number (D, F) and composition (E, G) in 3-month-old *GFRα1-CreER*^{T2} (D, E) and 12-month-old *GFRα1-EGFP* (F, G) mouse testes. Average ± SEM (n=3), *p>0.05, Student's *t* test. **(H, I)** Hematoxylin/Eosin stained section of 12-month-old *wild-type* (H) and *GFRα1-EGFP* (I) testis, suggesting that *GFRα1* heterozygosity does not affect the overall integrity of spermatogenesis. Inset: higher magnified image of seminiferous tubule. Bars are 100µm.

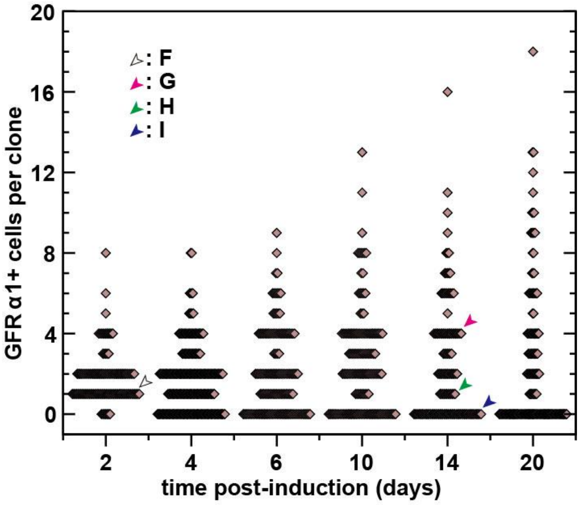

Fig. S2

Fig. S2: Supplemental data for fate analyses of pulse-labeled GFRα1+ spermatogonia clones in steady-state, related to Fig.2

Evolution of pulse-labeled clone size indexed by the number of GFRα1+ cells over 20 days. Data were obtained from 3, 4, 5, 4, 4, 6 and 3 testes at 2, 4, 6, 10, 14, and 20 days post-induction, respectively, summarized from the original data shown in Table S1. The clones shown in Fig. 2F-I are plotted as shown by white, magenta, green, and blue arrowheads, respectively.

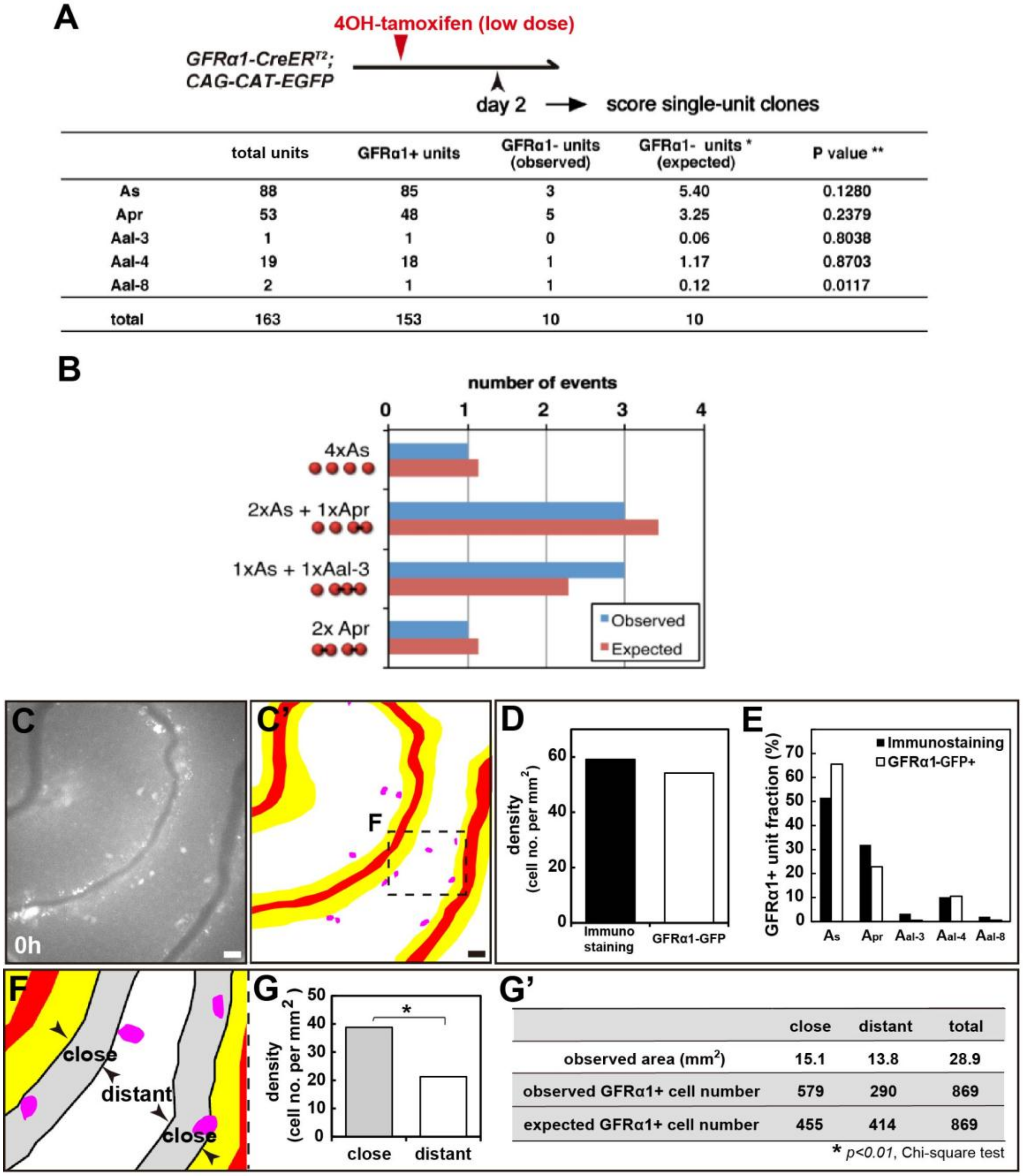

	total units	GFRα1+ units	GFRα1- units (observed)	GFRα1- units * (expected)	P value **
As	88	85	3	5.40	0.1280
Apr	53	48	5	3.25	0.2379
Aal-3	1	1	0	0.06	0.8038
Aal-4	19	18	1	1.17	0.8703
Aal-8	2	1	1	0.12	0.0117
total	163	153	10	10	

	close	distant	total
observed area (mm^2)	15.1	13.8	28.9
observed GFRα1+ cell number	579	290	869
expected GFRα1+ cell number	455	414	869

* $p<0.01$, Chi-square test

Fig. S3

Fig. S3: Supplemental data for live-imaging analyses of GFRα1+ spermatogonia, related to Fig.3

(A) Transition of GFRα1+ A_s, A_{pr} and A_{al} spermatogonia into GFRα1– cells, analyzed by short-term pulse-labeling fate analysis. As shown on the top, GFRα1+ spermatogonia were sparsely labeled in 3-month-old mice. 2 days later, induced clones were analyzed for GFRα1 expression, and those comprising a single spermatogonial unit were scored. The composition of pulse-labeled GFRα1– units roughly reflects that of spermatogonia that underwent GFRα1+ → Ngn3+ transition, while this could be biased to longer units to some extent because some of them may have passed a cell division that doubled the syncytial length after transition to Ngn3+ in this short period. Therefore, this result indicates that differentiation occurs in all the entities of spermatogonial units. *Expected numbers of GFRα1– units are calculated by dividing the total number of GFRα1– units (viz. ten) according to the proportion of the total GFRα1+ units, assuming that all the GFRα1+ units had the same probability to transit into GFRα1–, and that GFRα1– units did not divide after losing GFRα1 expression. **P-values were obtained by Chi-square test between observed and expected numbers of GFRα1– units for each entity compared with all of the other units. Counts from 6 testis samples were summarized. **(B)** Fragmentation patterns of GFRα1+ $A_{al\text{-}4}$ syncytia: In live-imaging study, all the possible patterns of fragmentation were observed at the indicated frequencies as shown in Fig. 3A (blue), which show a nice agreement with predicted values (red) (p=0.958 in Chi-square test). This prediction is done based on the assumption that each of the three intercellular bridges independently has 50% probability to break in an $A_{al\text{-}4}$ syncytium that is licensed to fragment. In this case, out of seven possible permutations in which at least one intercellular bridge break, 1, 3, 2, and 1 cases give rise to 4xA_s, 2xA_s + 1xA_{pr}, 1xA_s + 1x $A_{al\text{-}3}$, 2xA_{pr}, respectively. The expected frequencies are provided by allocation of the total 8 events according to these probabilities. **(C-E)** Density and composition of GFRα1-EGFP+ units observed by live-imaging in the surface area of mouse testes, compared with that of the tissue averages based on the whole-mount immunostained untangled seminiferous tubules. (C, C') Representative image obtained from the testis surface of *GFRα1-EGFP* mice (the first frame of Movie S4). (C') indicates the position of GFRα1+ spermatogonia (magenta), blood vessels (red) and interstitium (yellow) (C and C' are from the same image used in Fig.3E). (D, E) GFRα1+ cell densities (D) and unit fractions (E) counted by whole-mount immunostaining (black bars, data are reproduced from Fig.1D) and GFRα1-GFP fluorescence in seminiferous tubules located in surface area of *GFRα1-GFP* mouse testes (white bars). Counts from 3 testis samples were summarized. **(F-G')** Preferential localization of GFRα1+ spermatogonia near vasculature. (F) High magnification of the rectangular area in (C'), showing seminiferous tubule area divided into regions close to (<40μm; gray) and distant (>40μm; white) from the border of interstitium. (G) Density of GFRα1+ spermatogonia in regions close to and distant from the vasculature. Total density of GFRα1+ cells observed in 3 testes is shown. (G') Count of GFRα1-GFP+ cells observed in areas close to and distant from interstitium were significantly different from the expected distributions on the assumption of non-

biased distribution (*p<0.01, Chi-square test). Counts from 3 testis samples were summarized.

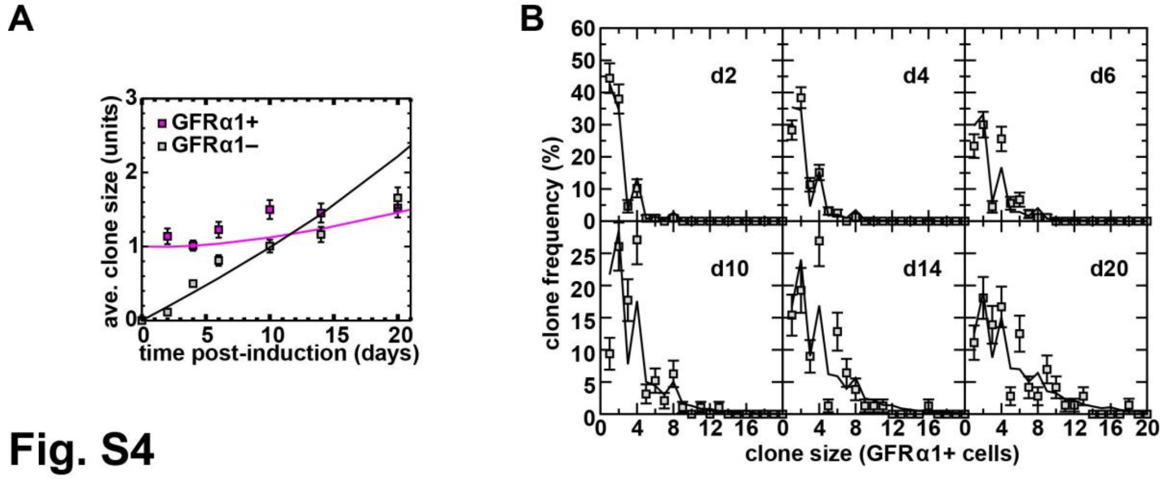

Fig. S4

Fig. S4: Supplemental data for model prediction of clonal fate behavior of GFRα1+ cells in steady state, related to Fig.4

(A) Capture of the average number of GFRα1+ (magenta) and GFRα1– (gray) units from the total clones observed *in vivo* (squares, average ± SEM among testes) by the model prediction (solid lines). **(B)** Capture of the distribution of GFRα1+ cell number in individual pulse-labeled clones scored *in vivo* (squares, average ± SEM among testes) by that predicted *in silico* (solid lines). *In vivo* data are reproduced from Fig.2L and Fig.S2, respectively.

A

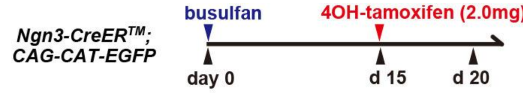

% labeling efficiency of Ngn3+ cells (a)	% contribution of labeled cells in GFRα1+ cells (b)	% contribution of Ngn3+ cells in GFRα1+ cells (c)	No. of GFRα1+ cells per mm tubules (d)	% contribution of Ngn3+ cells to steady-state value of GFRα1+ spermatogonia (e)
17.4 ± 6.5 ***	1.8 ± 0.3 ***	15.6 ± 5.3 ***	11.3 ± 0.9 ***	11.2 ± 4.7 ***

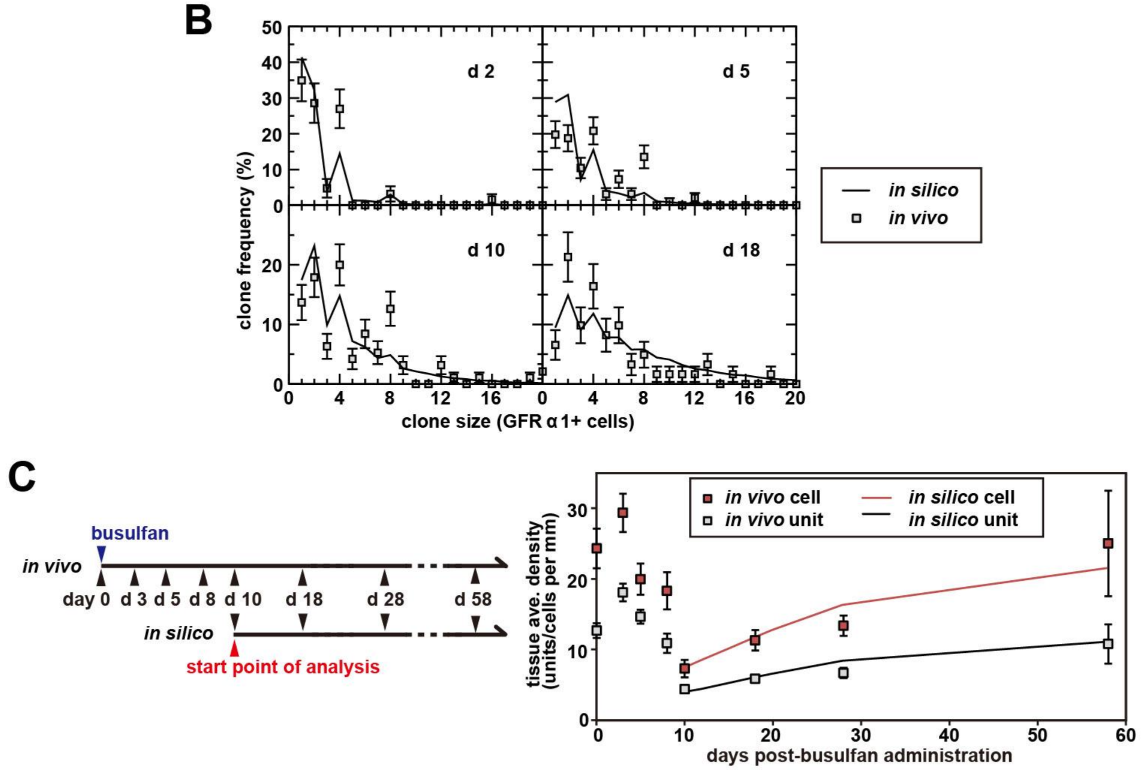

Fig.S5

Fig. S5: Supplemental data for the fate behavior of GFRα1+ spermatogonia in regeneration, related to Fig.6

(A) Contribution of Ngn3+ spermatogonia into the GFRα1+ compartment during the period of regeneration. As shown on the top, by using *Ngn3-CreER^{TM}; CAG-CAT-EGFP* mice, Ngn3+ cells were labeled by 4OH-tamoxifen administration during the regeneration process 15 days after busulfan injection, followed by the analyses of labeled cells 5 days later. (a) Labeling efficiency of Ngn3+ cells was estimated as the contribution of labeled cells to Kit+ differentiating spermatogonia observed in stages IX-XI on day 20, which had differentiated from undifferentiated spermatogonia that were Ngn3+ at the time of 4OH-tamoxofen administration. (b) Contribution of labeled cells to the total GFRα1+ spermatogonia on day 20. (c) Estimated contribution of cells that were Ngn3+ on day 15 to the GFRα1+ population on day 20 was calculated as (b)/(a). (d) Number of total GFRα1+ cells per mm tubules. (e) Contribution of Ngn3+ cells to the steady-state density of GFRα1+ cells (17 units/mm) during the 5 days, calculated

(c)*(d)/17. Numbers indicate average ± SEM from 4 testes. **(B)** Clone size distribution indexed by the number of GFRα1+ cells in individual clones in regeneration by the model using optimized parameters. As in Fig. 6, experimental data (squares) are shown as average ± SEM. *In silico* predictions are shown by solid lines. **(C)** Evolution of tissue average density of GFRα1+ spermatogonia during regeneration over two months. Left panel shows the time-schedule of *in vivo* data acquisition after busulfan administration (designated as day 0) and *in silico* analysis. Right panel shows the measurements of the tissue average density of GFRα1+ units/cells observed *in vivo* (squares) and the *in silico* model prediction (lines). Experimental data are reproduced form Nakagawa et al., 2010 and shown as average ± SEM from 3 testes at each time-point.

2. Supplemental Experimental Procedures

2-1. Intravital live imaging and image analysis

Live-imaging of the testes of four- to five-month-old *GFRα1*^{EGFP} or *GFRα1*^{EGFP}*;GATA1-EGFP* mice under anesthesia was performed for three days as described before, using epifluorescence IX61WI microscope (Olympus) (Yoshida et al., 2007). Time-lapse images were captured at the rate of one flame per 30 minutes using the Andor iXon EM-CCD camera controlled by Metamorph software (Molecular Devices). Movies were constructed by Metamorph software, and the trajectories of spermatogonia and Sertoli cells were manually extracted from the movies using the Metamorph and ImageJ softwares.

To classify complete and incomplete cell divisions, an intercellular bridge was deemed to be intact if the cells remained within 30µm for more than 12 hours, on the basis of the following observations. In the live-imaging study, we observed 35 divisions of A_s cells in total. In 4 cases, the resultant daughter cells subsequently separated into two A_s cells by the end of the record, whereas the daughter cells remained associated until the end of the record in the other 31 cases. The intervals between the division of parental A_s cell and the separation of the daughters in these 4 cases were 4, 11, 37, and 43 hours. Accordingly, we practically defined an A_s division as a "complete division", when the separation of the daughter cells were observed within 12 hours following division. So, the two cases with 4 and 11 hours of intervals were classified as complete divisions, while we categorized the other two cases (with 37 and 43 hours of intervals) as sequences of incomplete division followed by syncytial fragmentation. We set such a strict criterion so that we could avoid miscategorizing a complete division as "false incomplete division", while "false complete division" could be accepted to some extent (such as the one with 11 hours of interval after division).

2-2. Biophysical model

To explore and interpret the clonal dynamics of the GFRα1+ population, we developed a minimal biophysical modeling scheme, as described concisely in the main text. In this supplemental section, we set out in more detail the basis of the model and its practical implementation.

1) Framework of the modeling scheme

In accordance with the literature (Nakagawa et al., 2010; Sada et al., 2009; Yoshida, 2012), the result of the pulse-labeling experiment in the current study suggests that the population of GFRα1+ spermatogonia is primarily responsible for the stem cell activity (Fig. 2). In addition, the current live-imaging study revealed that GFRα1+ spermatogonia continually and reversibly interconvert between different state of A_s, A_{pr} and various lengths of A_{al} spermatogonia (Fig. 3). Therefore, we developed a biophysical modeling

scheme based on the assumption that stem cell activity was restricted to, and defined by the entirety of, the pool of morphologically heterogeneous GFRα1+ spermatogonia. In the model, for simplicity, the GFRα1+ → Ngn3+ transition was considered irreversible resulting in the permanent loss of GFRα1+ cells towards differentiation. Indeed, although the Ngn3+→GFRα1+ reversion has been observed, the preceding (as well as the current) studies showed that the frequency of this process is very low in steady state (Nakagawa et al., 2010, and Fig. 2D). In any case, taking into account the small fraction of Ngn3+ cells that transit back into the GFRα1+ compartment would not significantly affect the quantitative predictions of the model.

The GFRα1+ spermatogonial units are shown to be dispersed and actively moving over the tubules. Intriguingly, however, their local density, averaged over several millimeters of tubule, was found to be reproducibly constant. To reflect this situation, we proposed a regular quasi one-dimensional lattice model, in which each domain accommodates a single GFRα1+ unit, providing a caricature of the basal compartment of the seminiferous tubule (Fig. 4A). The lattice spacing along the longitudinal axis and around the circumference (1/3 mm in length and 1/5 of circumference) was chosen to be commensurate with the measured average density and their preferential localization to the vasculature (see main text). In this scheme, the territories of individual GFRα1+ units, which *in vivo* should be variable and even dynamic in their shape, area and arrangement, are considered even and regular in the tubules. Although this assumption of a regular lattice geometry is an obvious over-simplification, features sensitive to this approximation are expected to be small and beyond the resolution of our analysis.

Alongside the generation of Ngn3+ cells, the live-imaging study (Fig. 3A) showed that GFRα1+ units may undergo two further distinct processes: they may extend in unit length through incomplete cell division (Fig. 4B), and they may undergo multiplication through syncytial fragmentation (Fig. 4C). Based on the results of the live-imaging study (Fig. 3A), we supposed that the contribution made by the process of complete A_S cell division is sufficiently small that it may be neglected. Similarly, for simplicity, we neglected the apparently small contribution made by cell death of GFRα1+ cells.

In this scheme, to maintain the stem cell pool size (viz. the density of GFRα1+ units), the syncytial fragmentation (which multiplies GFRα1+ units) was correlated with the loss of a GFRα1+ unit (through transition to a Ngn3+ unit) on a neighboring domain. Practically, we supposed that, following fragmentation, one of the fragments stays at the "mother" site, while the others migrate to any one of the neighboring sites, where the existing GFRα1+ unit is lost through becoming Ngn3+ at that site.

2) Defining the constituent cell behavior

Even within the scope of such a simplified modeling scheme, we can contemplate numerous potential sources of variability that would increase the complexity of the model, in relation to the aforementioned cellular processes (viz. cell division, syncytial

fragmentation, and transition into being Ngn3+). For example, within the GFRα1+ population, the rates of any of these processes may depend sensitively on syncytial length. Similarly, syncytial fragmentation may cause the binary fission of a syncytium due to the breakage of a single intercellular bridge, or it may fragment into three or more components following the breaking of multiple bridges. Moreover, the fragmentation frequency may depend on the relative position of the bridge within the syncytium itself (which is related to the seniority of the bridge since its generation by incomplete cell division). Furthermore, the migration of syncytia between sites may be spatially anisotropic and it may involve transfer of syncytia over multiple lattice spacings. In addition, the behavior of cells after becoming GFRα1– may be correlated with the differentiation steps (Ngn3+, Kit+, or more advanced cell types).

Fortunately, we could take advantage of the observations from the live-imaging study to constrain the variability of the aforementioned cellular dynamics, which subsequently reduces the multitude of parameters *in silico*. In doing so, we found that the complex clonal dynamics observed *in vivo* conforms to a remarkably simple paradigm of the modeling scheme, as seen below.

First, from the results of the live-imaging study, it was apparent that the average GFRα1+ cell division rate, *D*, of approximately once per 10 days, is largely independent of the unit length (viz. regardless of being A_s, A_{pr} or A_{al}) (Fig. 3A). Second, although the frequency of fragmentation of GFRα1+ syncytia appeared to increase with unit size, the observed dependence could be captured by assuming that the rate correlates linearly with the number of intercellular bridges, which enabled the setting of a single rate constant of syncytial fragmentation (*F*) at once per 20 days per intercellular bridge. Moreover, the live-imaging data also suggested that any one of the bridges may break with around a 50% probability, once a syncytium is licensed to fragment (Fig. 3A, Fig. S3B).

Although the live-imaging data constrains the average rates of cell division and fragmentation as described above, the distribution of timings between the same or different events is, as yet, unspecified. In the following, for simplicity (to minimize the number of parameters), we assumed a Markov process in which the timings between consecutive events (cell division and fragmentation) were stochastic and statistically uncorrelated. In particular, these processes were drawn at random from an exponential distribution (Poisson process), with the defined average rates. While such an assumption was convenient, features associated with any degree of correlation between these processes, which may be present *in vivo*, would be rapidly erased from the clonal records.

Recognizing the role of the vasculature in specifying a facultative niche environment (Yoshida et al., 2007), we further assumed that some 70% of unit replacements occur along the axis of the tubule, while only 30% involve transfer across the tubule. Although this bias acknowledges the role of the vasculature in guiding the movement of GFRα1+ spermatogonia along the tubule axis, the properties of the model do not depend sensitively on the precise ratio. Further, based on the observed motility

of spermatogonial units in live-imaging (Fig. 3E-H), we allowed approximately 1/7 of replacements along to the tubule direction to occur at next-nearest neighbor sites. Once again, the model dynamics depends only weakly on this parameter.

Finally, the literature (Huckins and Oakberg, 1978) and our unpublished live-imaging observations indicated that GFRα1– (Ngn3+ and Kit+) units die to some degree. It is clear, however, that the death of GFRα1– units is not a stochastic event but preferentially observed in particular cell types that appear during the differentiation process. Nevertheless, since this event does not affect the dynamics of the surviving GFRα1+ population per se, we supposed that the death of GFRα1– cells also follow a Poisson process with a defined rate. Practically, we chose a death rate of around once per 30 days, which was consistent with the observations.

On extrapolating this model to predict longer-term clonal behavior over months to over a year, which was based on the measured clone length along the tubule axis and the number of the surviving clonal patches, it was supposed the differentiating cohort of GFRα1+ spermatogonia fully occupy domains of the lattice (which corresponds to the territory of an individual GFRα1+ unit) after a delay of approximately two weeks, during which time they mitotically amplify and reach the stage of spermatocytes. To reflect this, *in silico*, the length and number of the patches were predicted based on the occupation of domains by GFRα1+ spermatogonia two weeks in advance.

3) Characterization of the model dynamics and prediction of the *in vivo* observation

Despite the involvement of multiple components in this scheme, the dynamics of clonal evolution is essentially specified by just two parameters: the cell division rate, D, and the syncytial fragmentation rate per intercellular bridge, F. Moreover, in steady-state, the unit composition depends only on the ratio of these parameters, D/F. To investigate the clonal dynamics predicted by the model, one could consider the development of an analytical approach based on the analysis of a Master equation describing the time-evolution of the clone probability distribution (see, e.g., Lopez-Garcia et al., 2010). However, as a quasi one-dimensional system involving multiple components, the resulting Master equation would be analytically intractable. Fortunately, however, the steady-state unit composition and clone size distribution, both of which were independent measurements from those used to build up the model, could be recovered accurately from a straightforward numerical stochastic simulation of the dynamics of GFRα1+ spermatogonia under the foregoing modeling scheme.

Operationally, to follow the clonal dynamics of the GFRα1+ cell population, we began by seeding the lattice with a random configuration of GFRα1+ units in proportions that reflect the measured unit composition. To clonally trace these units, each was given a unique "barcode" which was then inherited by their progeny. The system was then allowed to evolve according to the dynamics specified above. As GFRα1+ units were lost through transition into being Ngn3+ and replaced following syncytial fragmentation, some clones that survived expanded while others became extinct by losing all the

GFRα1+ units. When GFRα1+ units were lost and became GFRα1–, a record was kept of their unit number on each domain. We did not, however, attempt to follow the expansion of GFRα1– units through cell division.

According to the defined dynamics, the model will converge from any arbitrary initial composition of spermatogonial units to a particular steady-state composition, which depends uniquely on the ratio of the cell division to fragmentation rates, D/F (Fig. 4D). Using the D and F rates of once per 10 days and once per 20 days per bridge, respectively, obtained directly from the live-imaging study (Fig. 3A), the model could predict the composition of the observed steady-state average of the GFRα1+ units with remarkable accuracy (Figs. 4D, E). We then used the computational scheme to predict the steady-state clonal evolution over 20 days following pulse-labeling. Comparison of the *in silico* prediction with the wide range of *in vivo* clonal fate data indexed by the number of GFRα1+ and GFRα1– spermatogonia within a clone revealed a surprisingly good agreement for both unit and cell number, over the entire 20 day time course (Figs.4G,H,I and Figs.S4A,B). Considering that the steady-state composition of GFRα1+ units and the clonal fates of pulse-labeled GFRα1+ units are totally independent of the data that were used to build up the modeling scheme, these agreements strongly support the validity of the modeling scheme. In addition, the validity of the model was further supported by its ability to predict the long-term (for months to over a year) clonal fates, and the dynamics in regeneration, as described in depth in the main text (Figs. 5, 6 and S5BC).

It is interesting to note that, by correlating syncytial fragmentation with the loss of GFRα1+, in steady-state, the effective rate of GFRα1+→Ngn3+ transition must be equal, by definition, to the cell division rate (D). More precisely, over $1/D$ days (the average cell division period, corresponding to 10 days based on the current live-imaging study), the total cell number is precisely doubled. Given the steady-state dynamics, one half of these cells must exit the GFRα1+ compartment, while the other half remains GFRα1+ during this period. If the overall transition occurs in proportion to the steady-state composition of GFRα1+ units, the spermatogonia that have become GFRα1– over the $1/D$ days must be equal to that of the original GFRα1+ population, in both cell and unit numbers. Because transition to a GFRα1– unit is allied with replacement following a multiplication of the neighboring GFRα1+ unit, the above property also shows that the rate of replacement between neighboring domains should also be equal to the rate of cell division, or once per 10 days in particular. Of note, this agrees with the previous estimation of the replacement rate to be once per less than two weeks based on the long-term (months to over a year) fate analyses of Ngn3+ spermatogonia-derived patches (Klein et al., 2010).

The current model also recovers the scaling function, a hallmark behavior of the population asymmetry, which was observed in the long-term distribution of the clone size indexed by patch length (Fig. 5D)(Klein et al., 2010). These findings not only support the premise that the observed long-term behavior reflected that of GFRα1+ spermatogonia to which the labeled Ngn3+ cells were expected to revert, but also provide a cell-level explanation of the ongoing stochastic stem cell loss and replacement.

4) Consideration of alternative scenarios

Although the results in this study fully support the theory of single stem cell pool composed of functionally equivalent GFRα1+ A_s and syncytia, as implied by the proposed model (Fig. 7A), one may also conceive of alternative scenarios. For example, a small compartment of slow-cycling GFRα1+ A_s cells that act as the “true” stem cells might undergo rare asymmetrical division (an infrequent event corresponding to the low number of complete divisions that were observed). Then, one of the daughter A_s cells could replenish and maintain the stock of slow-cycling stem (A_s) cells. The other daughter A_s GFRα1+ cell would then transfer to the active GFRα1+ compartment. This second compartment, which is much larger than the former in number, would repeat incomplete division and syncytial fragmentation as observed in this study. However, cells in the second, active, compartment would have limited short-term longevity and, eventually, would become replaced by the A_s daughter from the slow cycling compartment to maintain long-term homeostasis. As described below, although we don’t –and can’t– rigorously rule out the presence of such a slow-cycling compartment, we can conclude that their contribution (would they exist) would not be essential for the maintenance of life-long spermatogenesis in mouse.

Indeed, the “equipotent model” (that proposed in this study) is by itself able to provide a highly accurate quantitative prediction of the short-term clonal fate data over the 20 day time course. However, it would be difficult to rule out alternative models such that described above solely on the basis of these short-term data. However, even if the “slow-cycling” model could capture aspects of the “short-term” dynamics (which is far from clear), the “long-term” (which we defined here as a time scale from months to over a year, which effectively covers the reproduction period of the mouse) behavior of the pulse-labeled GFRα1+ spermatogonia provides a key for discrimination between these models.

Importantly, the “equipotent” model, which is synthesized solely from measurements in the live-imaging study (up to 3 days), quantitatively predicts not only the short-term (up to 20 days) but also the long-term (up to 14 months) clonal behavior (viz. continuous clonal loss and the size distribution of surviving clones: Fig. 5). Of particular note, the predicted long-term behavior does not require any adjustment of the model, but is achieved by its simple extrapolation to long times. In other words, both short- and long-term behaviors are fully explained by this same minimal model, and do not require other factors (such as a “slow-cycling” A_s population) to explain the wide range of experimental observations. In this context, it is important to understand that the equipotent model faithfully recovers the “scaling” behavior of the clone size distribution over different time scales, a robust and parameter-independent hallmark of stochastic stem cell loss and replacement (Klein and Simons, 2011).

On the other hand, if one assumes that tissue is maintained long-term by a “slow-cycling” A_s cell compartment, then we would see two characteristic behaviors at different time scales. In the short term (relative to the turnover time of the slow cycling

cells), we would see the clonal depletion and the expansion of the surviving clones, reflecting the feature of short-lived “active” GFRα1+ cells that are positioned inferior to the long-lived “slow-cycling” GFRα1+ cells and destined to disappear from the tissue. After this transient phase of loss of clones derived from short-lived cells, we would then expect a transfer of the clonal dynamics to a new distinct phase, where rates of clonal loss and replacement should significantly decrease reflecting the persistence of clones originating from the “slow-cycling” A_s cells. Contrary to this expectation, in reality, the observed fate behavior of GFRα1+ cells follows from a single dynamics (again, defined by just two rates measured from the very-short-term live-imaging study), in both short- and long-term. Therefore, the contribution of a slow-cycling stem cell population, which is presumably located on the top of the hierarchy, is not quantitatively supported by the data. In addition, on the basis of the same experimental and mathematical evidence described above, if the population of GFRα1+ spermatogonia involve multiple subsets characterized by different kinetics, significant contribution of any kind of slowly-turning-over population is not supported.

3. Supplemental Table S1

The entire clonal fate raw data of pulse-labeled GFRα1+ spermatogonia in steady state, related to Fig. 2 (provided as a separate Excel file). Data are summarized from 3, 4, 5, 4, 4, 6 and 3 testes for steady-state measure and at 2, 4, 6, 10, 14, and 20 days post-induction, respectively.

4. Supplemental Movies

Movie S1: An example of A_s →2x A_s division, supporting Fig. 3B
Movie S2: An example of A_s → A_{pr} division, supporting Fig. 3C
Movie S3: An example of cell division of A_{pr}→A_{al-4} followed by a fragmentation into an A_s and an A_{al-3}, supporting Fig. 3D
Movie S4: Prominent migration of GFRα1-EGFP+ spermatogonia, supporting Fig. 3E-F
Movie S5: Migration of GFRα1-EGFP+ spermatogonia between Sertoli cells revealed by in vivo live imaging of GFRα1-EGFP; GATA1-EGFP mouse testis, supporting Fig. 3G-H
Throughout, the time scale is shown as elapsed time in days: hours: minutes.

5. Supplemental References

Bunting, M., Bernstein, K.E., Greer, J.M., Capecchi, M.R., and Thomas, K.R. (1999). Targeting genes for self-excision in the germ line. Genes Dev *13*, 1524-1528.

Enomoto, H., Hughes, I., Golden, J., Baloh, R.H., Yonemura, S., Heuckeroth, R.O., Johnson, E.M., and Milbrandt, J. (2004). GFRalpha1 expression in cells lacking RET is dispensable for organogenesis and nerve regeneration. Neuron *44*, 623-636.

Lopez-Garcia, C., Klein, A.M., Simons, B.D., and Winton, D.J. (2010). Intestinal stem cell replacement follows a pattern of neutral drift. Science *330*, 822-825.
