# Supplementary figures and images for: Mouse Spermatogenic Stem Cells Continually Interconvert between Equipotent Singly Isolated and Syncytial States
Source: Cell Stem Cell. 2014 May 1;14(5):658–72. doi: 10.1016/j.stem.2014.01.019 (PMC4010676; doi:10.1016/j.stem.2014.01.019)

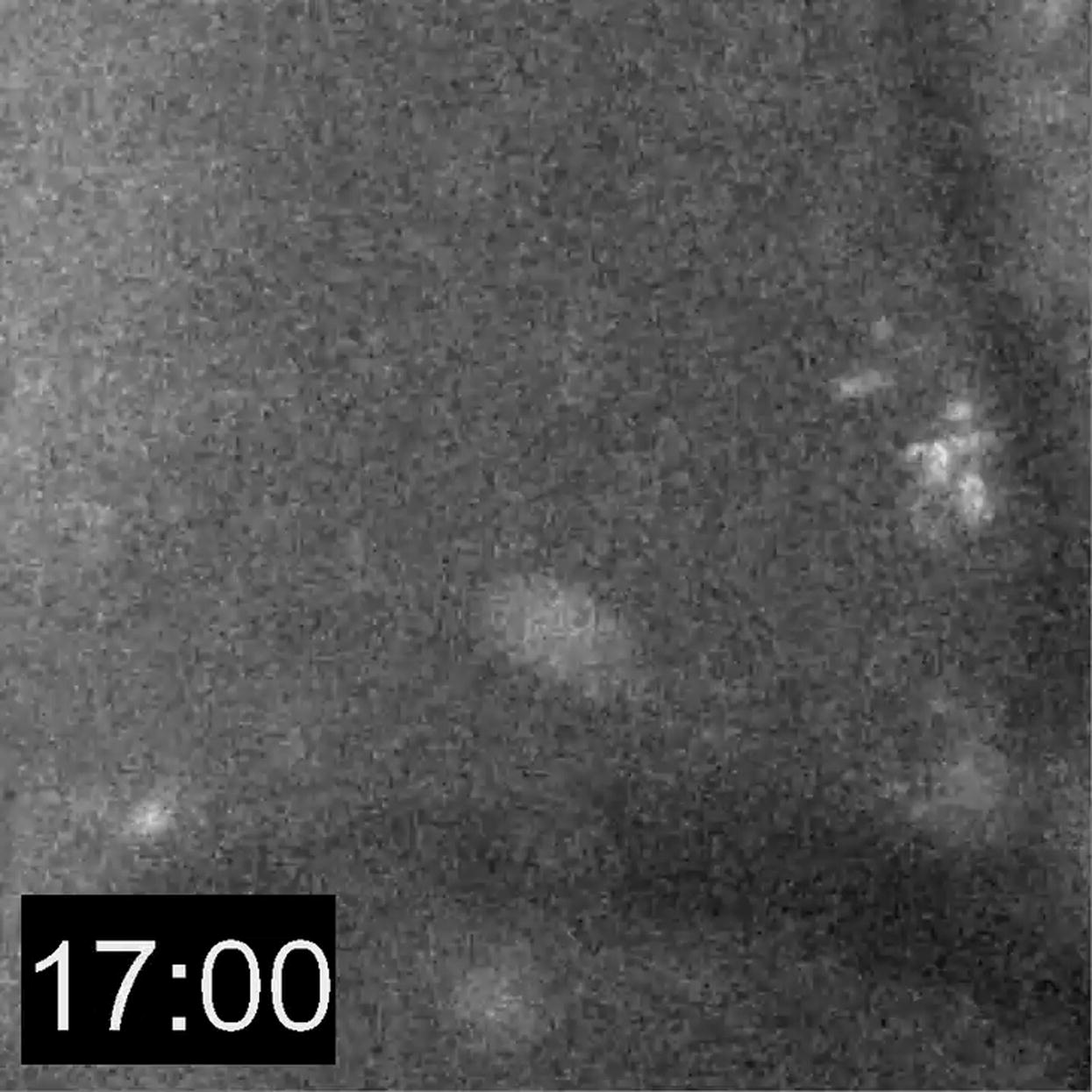

Supplement: Movie S1. An Example of As → 2 × As Division, Related to Figure 3B — The time scale is shown as elapsed time in days:hours:minutes. [file mmc3.jpg]

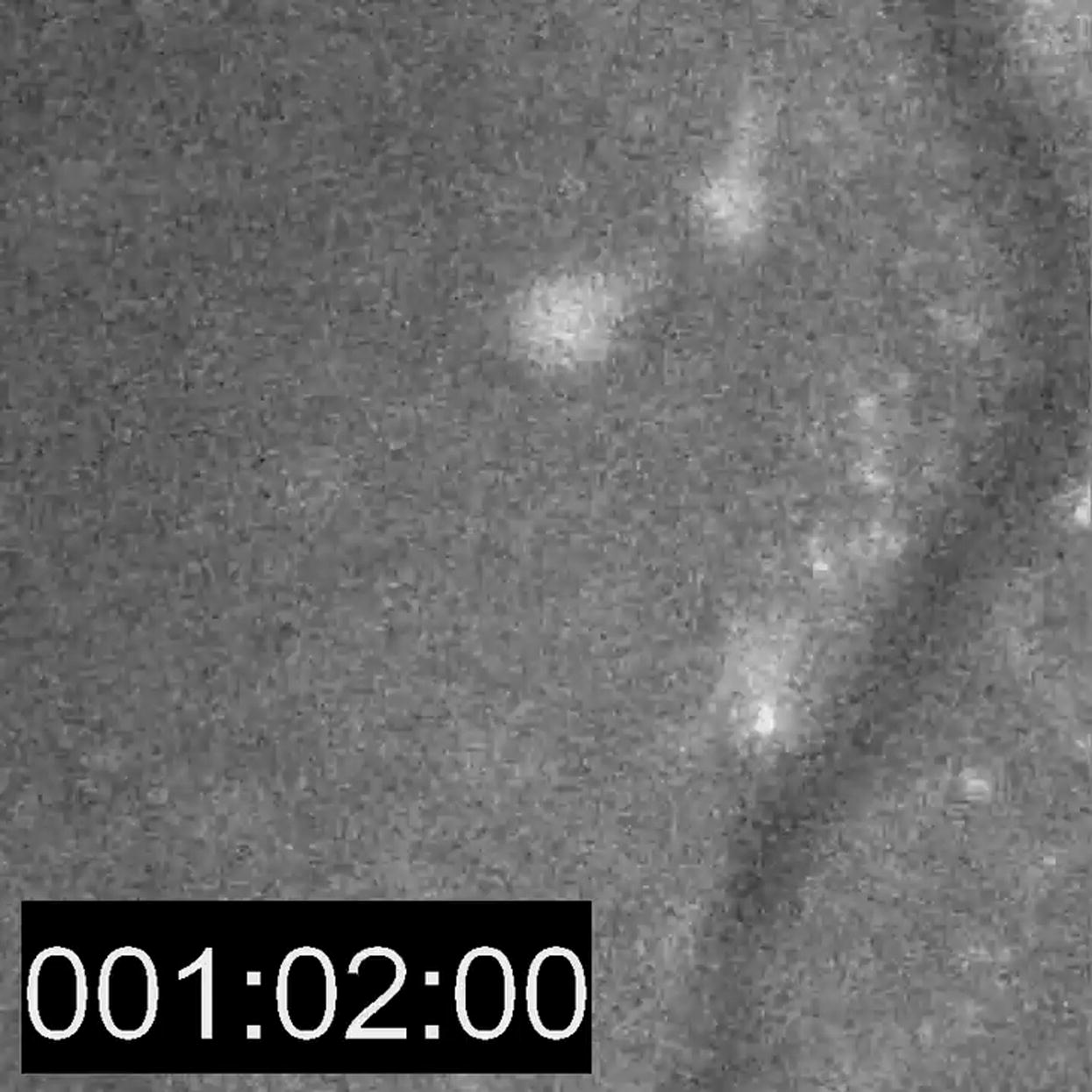

Supplement: Movie S2. An Example of As → Apr Division, Related to Figure 3C — The time scale is shown as elapsed time in days:hours:minutes. [file mmc4.jpg]

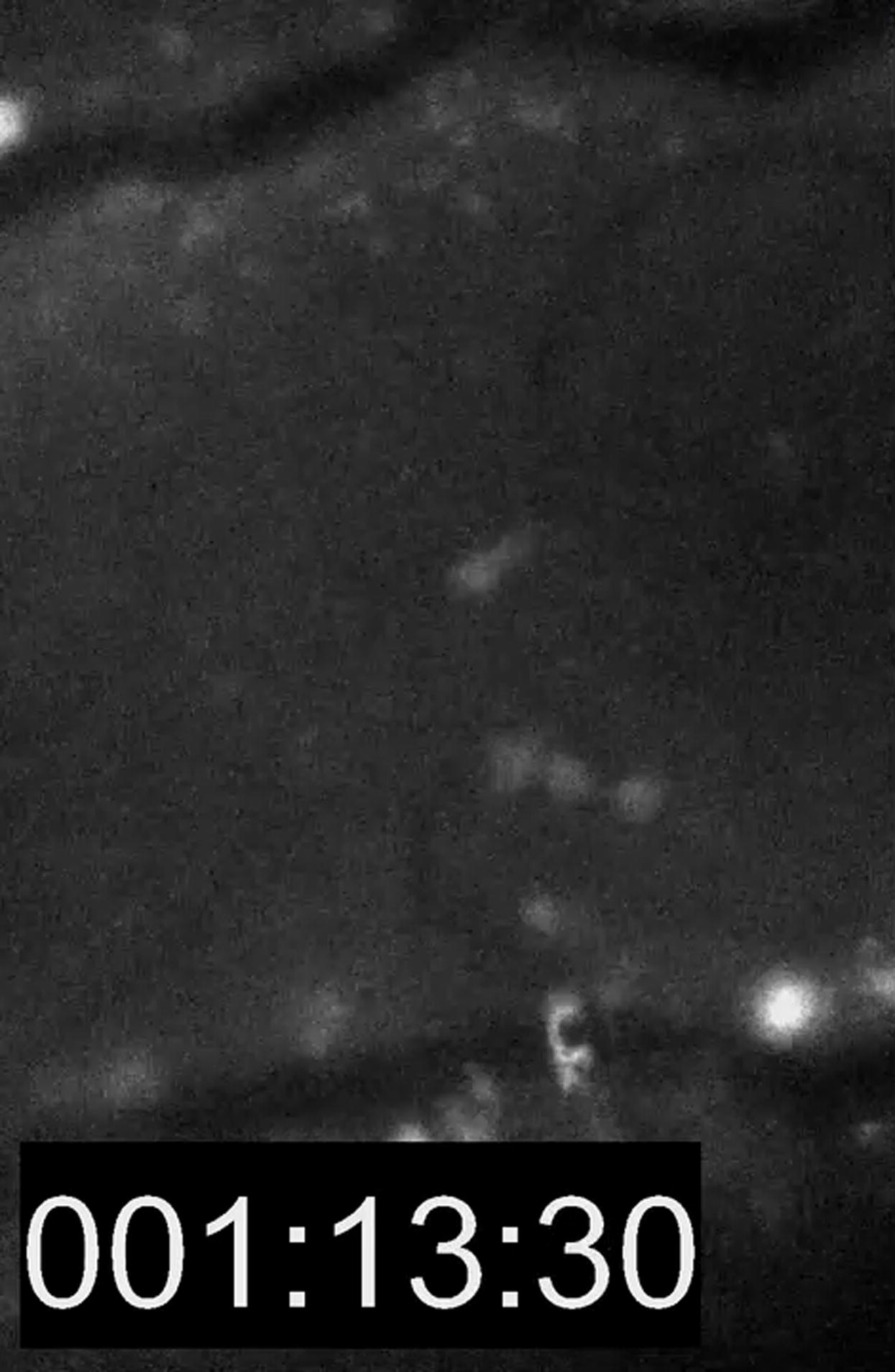

Supplement: Movie S3. An Example of Cell Division of Apr→Aal-4, followed by a Fragmentation into an As and an Aal-3, Related Figure 3D — The time scale is shown as elapsed time in days:hours:minutes. [file mmc5.jpg]

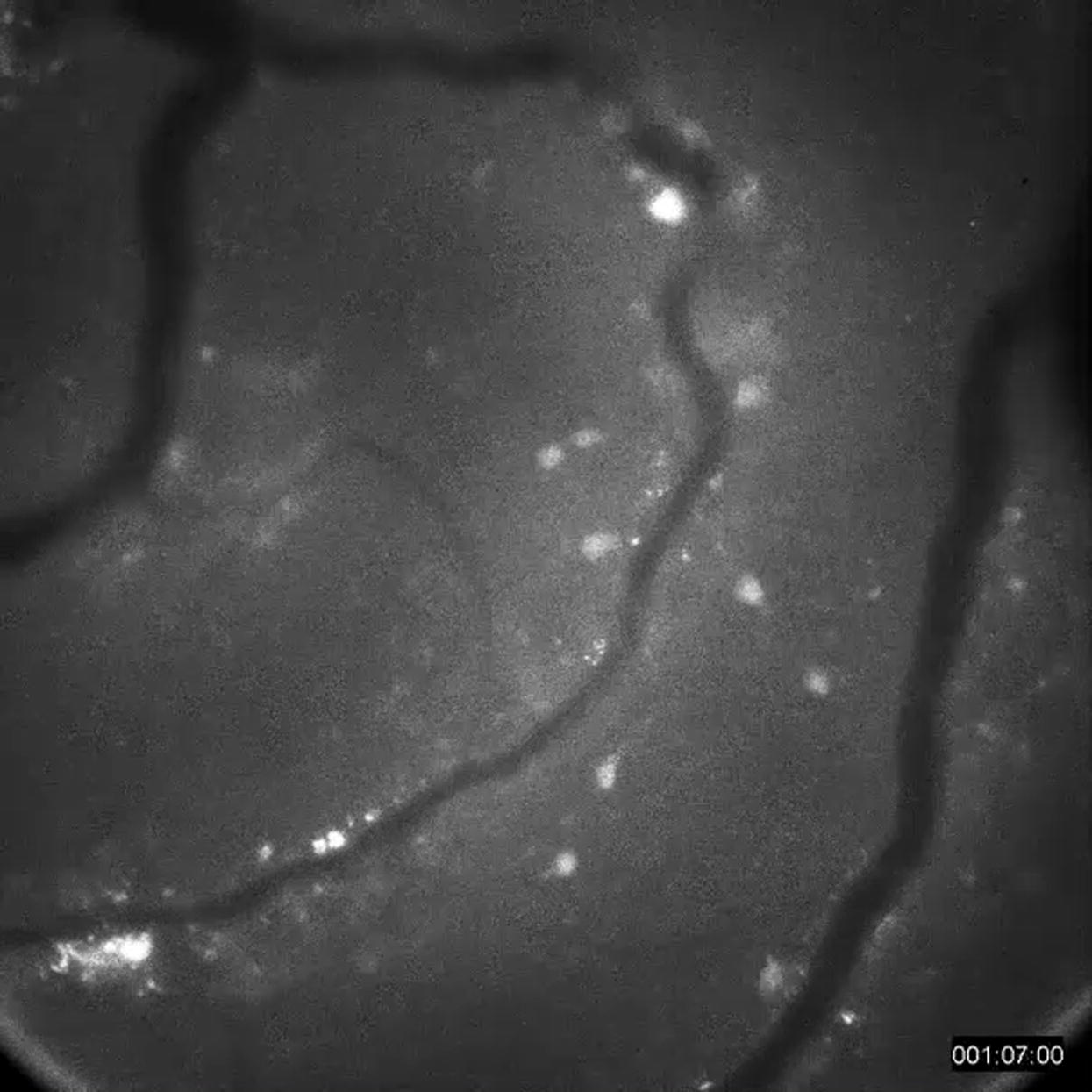

Supplement: Movie S4. Prominent Migration of GFRα1-EGFP+ Spermatogonia, Related to Figures 3E and 3F — The time scale is shown as elapsed time in days:hours:minutes. [file mmc6.jpg]

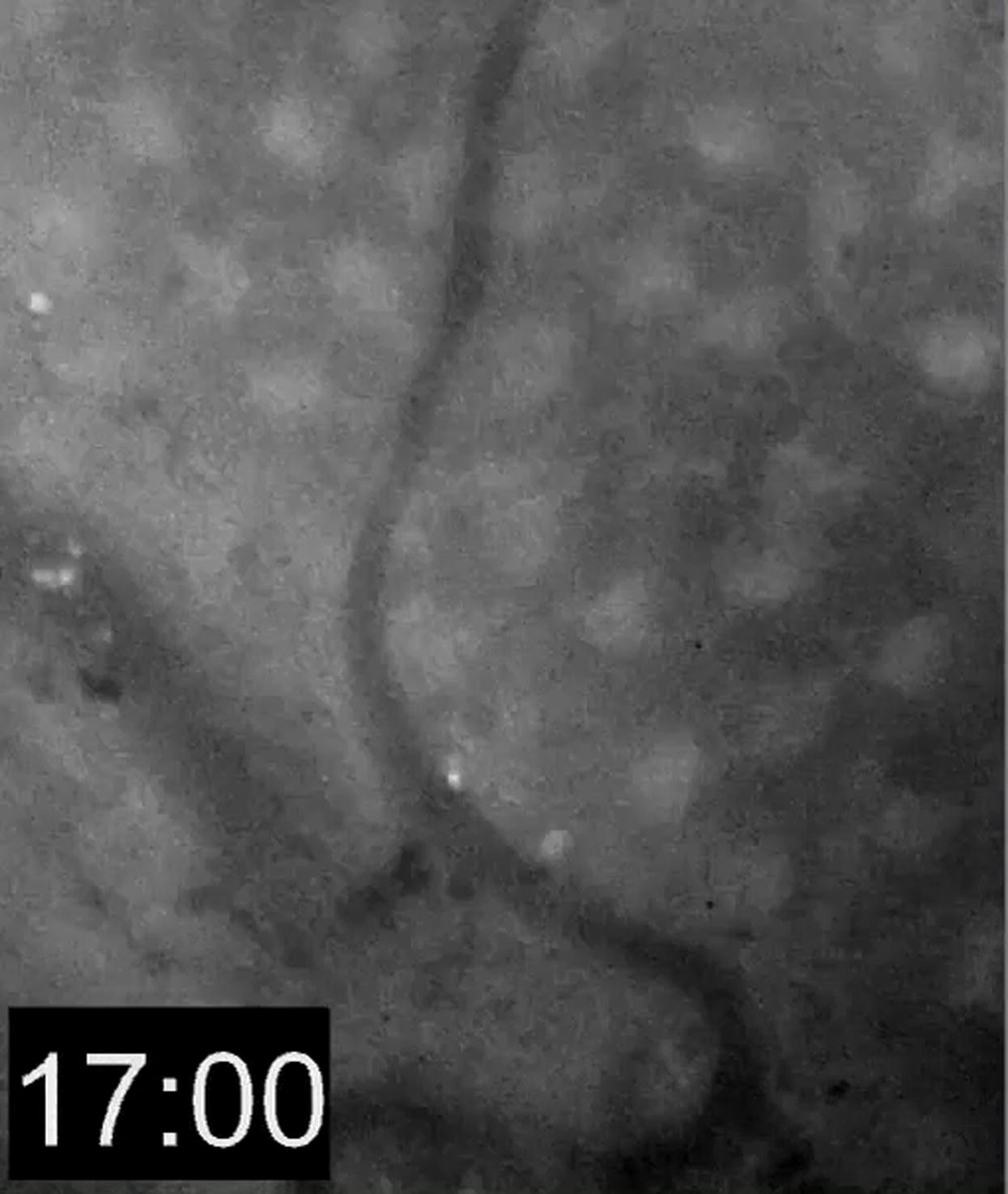

Supplement: Movie S5. Migration of GFRα1-EGFP+ Spermatogonia between Sertoli Cells Revealed by In Vivo Live Imaging of GFRα1-EGFP; GATA1-EGFP Mouse Testis, Related to Figures 3G and 3H — The time scale is shown as elapsed time in days:hours:minutes. [file mmc7.jpg]
