# Supplementary material for: Mouse Spermatogenic Stem Cells Continually Interconvert between Equipotent Singly Isolated and Syncytial States
Source: Cell Stem Cell. 2014 May 1;14(5):658–72. doi: 10.1016/j.stem.2014.01.019 (PMC4010676; doi:10.1016/j.stem.2014.01.019)
Supplement: Document S2. Article plus Supplemental Information [file mmc8.pdf]

Cell Stem Cell
Article

Mouse Spermatogenic Stem Cells Continually Interconvert between Equipotent Singly Isolated and Syncytial States

Kenshiro Hara,[1,2] Toshinori Nakagawa,[3] Hideki Enomoto,[4,5] Mikiko Suzuki,[6] Masayuki Yamamoto,[6] Benjamin D. Simons,[7,8,9,*] and Shosei Yoshida[1,2,*]
[1]Division of Germ Cell Biology, National Institute for Basic Biology, National Institutes of Natural Sciences, 5-1 Higashiyama, Myodaiji, Okazaki, 444-8787, Japan
[2]Department of Basic Biology, School of Life Science, Graduate University for Advanced Studies (Sokendai), 5-1 Higashiyama, Myodaiji, Okazaki, 444-8787, Japan
[3]Department of Immunobiology and Hematology, Institute for Frontier Medical Sciences, Kyoto University, 53 Kawahara-cho, Shogoin, Sakyo-ku, Kyoto, 606-8507, Japan
[4]Division of Neural Differentiation and Regeneration, Department of Physiology and Cell Biology, Graduate School of Medicine, Kobe University, 7-5-1 Kusunoki-cho, Chuo-ku, Kobe 650-0017, Japan
[5]Laboratory for Neuronal Differentiation and Regeneration, RIKEN Center for Developmental Biology, 2-2-3 Minatojima-Minamimachi, Chuo-ku, Kobe, 650-0047, Japan
[6]Department of Medical Biochemistry, Tohoku University Graduate School of Medicine, Aoba-ku, Sendai, 980-8575, Japan
[7]Cavendish Laboratory, Department of Physics, University of Cambridge, J.J. Thomson Avenue, Cambridge CB3 0HE, UK
[8]The Wellcome Trust/Cancer Research UK Gurdon Institute, University of Cambridge, Tennis Court Road, Cambridge CB2 1QN, UK
[9]Wellcome Trust-Medical Research Council Stem Cell Institute, University of Cambridge, Tennis Court Road, Cambridge CB2 1QR, UK
*Correspondence: bds10@cam.ac.uk (B.D.S.), shosei@nibb.ac.jp (S.Y.)
http://dx.doi.org/10.1016/j.stem.2014.01.019
This is an open access article under the CC BY license (http://creativecommons.org/licenses/by/3.0/).

SUMMARY

The identity and behavior of mouse spermatogenic stem cells have been a long-standing focus of interest. In the prevailing "A_s model," stem cell function is restricted to singly isolated (A_s) spermatogonia. By examining single-cell dynamics of GFRα1+ stem cells in vivo, we evaluate an alternative hypothesis that, through fragmentation, syncytial spermatogonia also contribute to stem cell function in homeostasis. We use live imaging and pulse labeling to quantitatively determine the fates of individual GFRα1+ cells and find that, during steady-state spermatogenesis, the entire GFRα1+ population comprises a single stem cell pool, in which cells continually interconvert between A_s and syncytial states. A minimal biophysical model, relying only on the rates of incomplete cell division and syncytial fragmentation, precisely predicts the stochastic fates of GFRα1+ cells during steady state and post-insult regeneration. Thus, our results define an alternative and dynamic model for spermatogenic stem cell function in the mouse testis.

INTRODUCTION

In mammalian testes, spermatogenic stem cells are responsible for both the continual production of sperm in steady state and regeneration following injury (de Rooij and Russell, 2000; Meistrich and Van Beek, 1993; Yoshida, 2012). However, the dynamics of the stem cell population remain largely unresolved at the single-cell level. The process of spermatogenesis takes place in seminiferous tubules (Figure 1A). All stages of germ cells are nourished by somatic Sertoli cells, which support a prominent network of tight junctions that separate the basal and adluminal compartments and, together with the basement membrane, provide the structural basis of the tubules. The tubules are surrounded by peritubular cells, whereas the intertubular space is made up of a network of blood vessels and interstitial cell types. Spermatogonia (mitotic germ cells that include stem cells) lie in close association with the basement membrane in the basal compartment. When meiosis begins, cells detach from the basement membrane and translocate across the tight junctions, after which they undergo meiotic divisions and spermiogenesis, and mature sperm are released into the lumen. This organization is observed uniformly throughout the entire 1.7 m tubule length that constitutes a single mouse testis (Russell et al., 1990), suggesting that seminiferous tubules lack a discrete anatomically defined niche.

In mouse, spermatogonia are divided into "undifferentiated" and "differentiating" populations (Figures 1A and 1B). "Undifferentiated spermatogonia" are found as singly isolated cells (A_s) or syncytia consisting mainly of 2 (A_{pr}), 4 ($A_{al\text{-}4}$), 8 ($A_{al\text{-}8}$), or 16 ($A_{al\text{-}16}$) cells. The formation of syncytia is due to "incomplete division," a germline-specific cell division process by which cytokinesis does not complete and cytoplasmic connection between daughter cells persists via intercellular bridges (de Rooij and Russell, 2000; Russell et al., 1990). This process continues through subsequent mitotic and meiotic divisions, resulting in the extension of syncytia from differentiating spermatogonia (A_1, A_2, A_3, A_4, Intermediate, and B) to haploid spermatids.

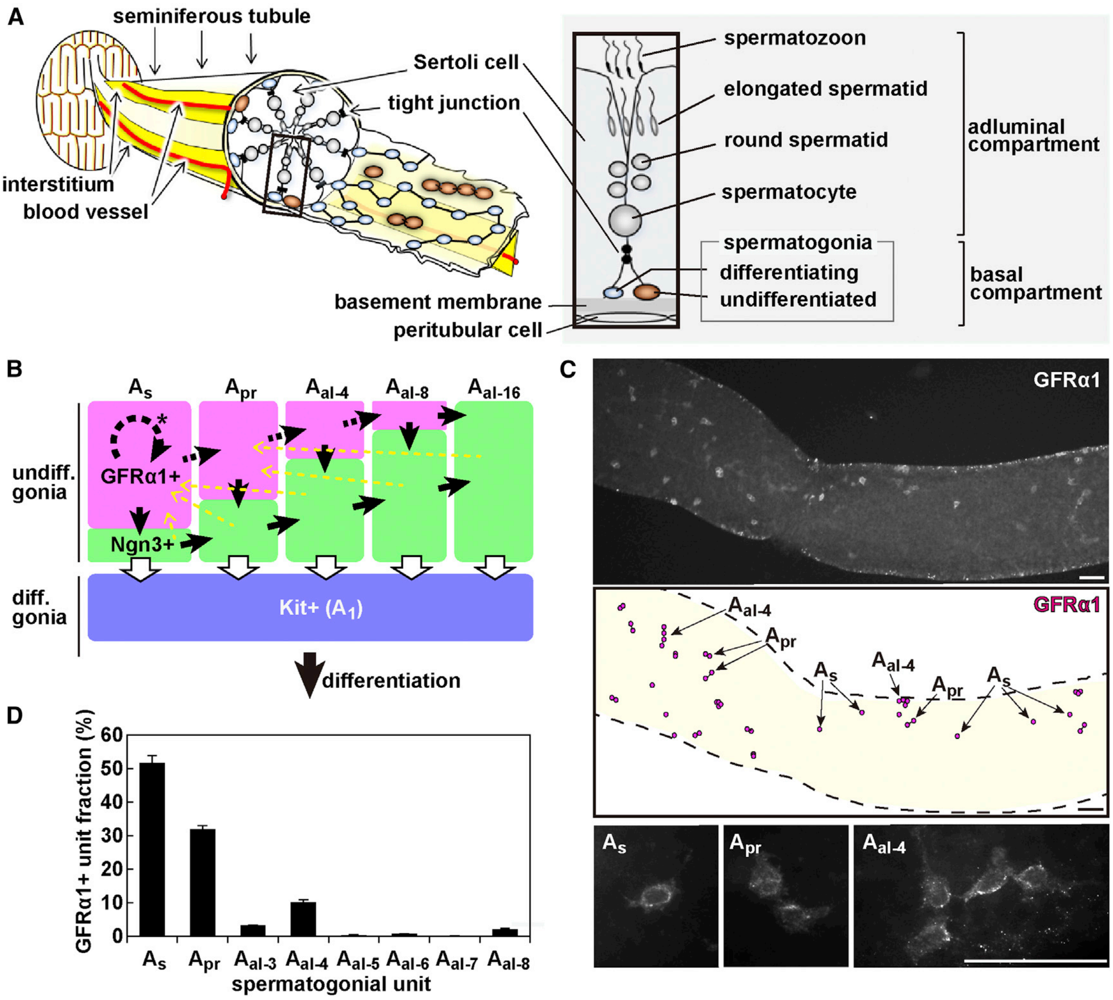

Figure 1. GFRα1+ Spermatogonia in Mouse Seminiferous Tubules

(A) Anatomy of seminiferous tubules. Undifferentiated spermatogonia (brown) and differentiating spermatogonia (blue) are distributed among Sertoli cells in the basal compartment (see text for details).

(B) A proposed hierarchy of GFRα1+ and Ngn3+ subpopulations of undifferentiated spermatogonia, as well as Kit+ differentiating spermatogonia (modified from Nakagawa et al., 2010). Black and white solid arrows indicate processes that have been directly observed, whereas the black broken arrows represent presumptive dynamics of GFRα1+ cells, in which only GFRα1+ A_s self-renew (asterisk). Yellow broken arrows indicate the processes of "reversion," which occur infrequently in steady state.

(C) Immunofluorescence for GFRα1 in whole-mount seminiferous tubule specimen. Middle panel: distribution of GFRα1+ spermatogonia. Lower panels: higher magnification of GFRα1+ A_s, A_{pr}, and A_{al-4}. Scale bars, 50 μm.

(D) Composition of GFRα1+ spermatogonial units observed in adult mouse testis. Averages ± SEM from three testes are shown.

Experimentally, posttransplantation colony-formation and regeneration assays established that, whereas Kit-positive (Kit+) differentiating spermatogonia seem to retain some stem cell potential, the vast majority of stem cell function is restricted to Kit-negative (Kit−) undifferentiated spermatogonia (Barroca et al., 2009; Ohbo et al., 2003; Shinohara et al., 2000). Based on the detailed analyses of fixed specimens, it was proposed in 1971 that stem cell activity may be restricted to the population of A_s spermatogonia, whereas interconnected A_{pr} and A_{al} syncytia are irreversibly committed to differentiation and no longer contribute to the stem cell pool (Huckins, 1971; Oakberg, 1971). This hypothesis, which has become the prevailing theory, is known as the "A_s model."

The population of undifferentiated spermatogonia is further divided according to their heterogeneous gene expression (Hofmann et al., 2005; Nakagawa et al., 2010; Sada et al., 2009; Suzuki et al., 2009a; Yoshida et al., 2004, Yoshida, 2012; Zheng et al., 2009). In undisturbed steady-state spermatogenesis, the GFRα1+ subpopulation (mainly A_s, A_{pr}, and fewer A_{al}) is thought to reside on the top of the hierarchy (Nakagawa et al., 2010; Sada et al., 2009). As well as maintaining their own population, GFRα1+ cells also give rise to the second, Ngn3+, subpopulation of undifferentiated spermatogonia (comprised of more A_{al} and fewer A_s and A_{pr}) (Figure 1B). Previous studies using live imaging and cre-mediated pulse labeling of Ngn3+ spermatogonia have demonstrated that the majority of

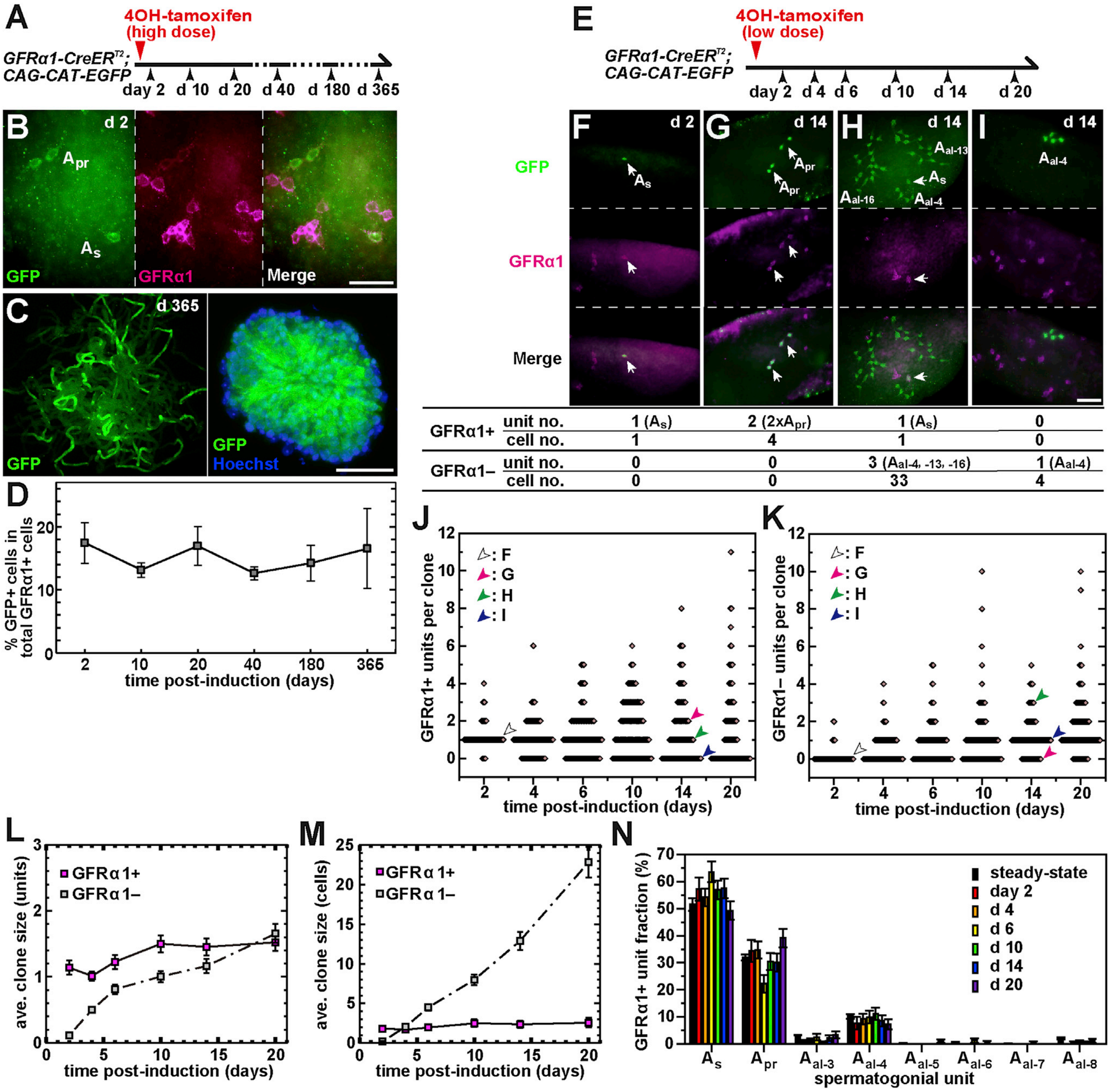

Figure 2. Pulse-Labeling Analyses of GFRα1+ Spermatogonia

(A) Experimental schedule for (B)–(D). *GFRα1-CreER^{T2}; CAG-CAT-EGFP* mice were administered with 2.0 mg 4OH-tamoxifen to pulse label GFRα1+ units with GFP, and their testes were analyzed at the indicated time points.

(B) Labeling of a fraction of GFRα1+ cells (magenta) with GFP expression (green) 2 days after pulse.

(C) Untangled seminiferous tubules at 365 days postlabeling, showing numerous patches of GFP+ cells (left) and a cross-section of such a patch in which GFP signal is found in all stages of germ cell differentiation (right).

(D) Fraction of GFP+ cells out of total GFRα1+ population from 2 to 365 days postinduction. Averages ± SEM from 3, 4, 4, 3, 3, and 3 testes for 2, 10, 20, 40, 180, and 365 days postinduction are shown, respectively.

(E) Experimental schedule for clonal fate analysis of pulse-labeled GFRα1+ units in (F)–(N). *GFRα1-CreER^{T2}; CAG-CAT-EGFP* mice were administrated with 0.35 mg 4OH-tamoxifen to sparsely label the GFRα1+ spermatogonia at an efficiency of 1.0% ± 0.1% (n = 3) and analyzed at the indicated time points.

(F–I) Whole-mount staining of seminiferous tubule for GFP (green) and GFRα1 (magenta) at 2 (F) and 14 (G, H, and I) days postinduction; stains are scored as shown below. Arrows indicate the labeled GFRα1+ units.

(J and K) Distribution of clone size as measured by GFRα1+ (J) and GFRα1− (K) unit number per clone over time. Each dot indicates one clone. The clones shown in (F)–(I) are plotted as shown by white, magenta, green, and blue arrowheads, respectively.

(legend continued on next page)

Ngn3+ cells transfer to the next Kit+ differentiating spermatogonia (Nakagawa et al., 2007, 2010). Intriguingly, these studies also revealed that Ngn3+ cells retain the capability of regaining GFRα1 expression, fragmenting into single cells or shorter syncytia (through breaking of intercellular bridges), and contributing to the long-term stem cell pool (Figure 1B, yellow dotted arrows), suggesting that the entirety of undifferentiated spermatogonia (both GFRα1+ and Ngn3+) may contribute to stem cell activity. However, such "reversion" of Ngn3+ cells takes place only infrequently in steady state but becomes prevalent in regeneration following tissue insult or transplantation. Therefore, Ngn3+ cells have been considered as a reserve population, whereas GFRα1+ cells are thought to represent the primary population responsible for the stem cell function (Nakagawa et al., 2007, 2010; Spradling and Fan, 2010).

Such flexible behavior of Ngn3+ cells, especially syncytial fragmentation, questions the premise of the "A_s model" that syncytia are irreversibly committed to differentiation. It was also shown that, in common with other Ngn3+ spermatogonia, Ngn3+ A_s cells are strongly biased toward differentiation to Kit+, indicating that not all the A_s spermatogonia function equally as stem cells (Nakagawa et al., 2010). In addition, based on the occasional contribution of Ngn3+ cells to the long-term stem cell pool, the behavior of the pulse-labeled stem cells was analyzed for months to over a year. The results demonstrated that stem cells are continually and stochastically lost and replaced by their neighbors, through a process of population asymmetry (Klein et al., 2010; Nakagawa et al., 2007). This observation not only further challenged the "A_s model" but also questioned the cellular basis of stem cell loss and replacement.

To summarize, the preceding analyses of how Ngn3+ spermatogonia behave over time (by means of pulse labeling and live imaging) have questioned the validity of the "A_s model," at least in its original form. Although A_s spermatogonia that are GFRα1+ were presumed to be the primary stem cell type (Nakagawa et al., 2010) (Figure 1B, black dotted arrow with asterisk), this conjecture lacks direct experimental support. To understand the stem cell dynamics, therefore, it is essential to dissect the fate behavior of GFRα1+ spermatogonia over time. In this study, we developed knockin mouse models and conducted intravital in vivo live-imaging and pulse-labeling studies of GFRα1+ spermatogonia at a single-cell resolution. On the basis of the unexpected behavior of GFRα1+ cells observed in these studies, we propose an alternative theory of the identity and behavior of mouse spermatogenic stem cells.

RESULTS

Stem Cell Function of GFRα1+ Spermatogonia

GFRα1+ spermatogonia lie scattered unevenly on the basement membrane of seminiferous tubules (Figures 1A and 1C). Intriguingly, however, their local density over a prolonged tubule length (several millimeters) was found to be remarkably constant in adult mice, with 17 ± 1 "units" of GFRα1+ spermatogonia per mm of tubule. Here, we define "a unit" as either "an A_s spermatogonium" or "a single syncytium consisting of multiple spermatogonia." Their composition was also conserved between individuals, not only for A_s, A_{pr}, and $A_{al\text{-}4}$, which comprised some 50%, 30%, and 10% of total units, respectively, but also for the small numbers of $A_{al\text{-}8}$ and "odd-sized" syncytia (e.g., 3-, 5-, and 6-cell chains designated hereafter as $A_{al\text{-}3}$, $A_{al\text{-}5}$, and $A_{al\text{-}6}$), which together constitute the remaining 10% (Figure 1D).

To trace the fate of GFRα1+ spermatogonia, we developed a knockin mouse model that enables the pulse labeling of GFRα1+ cells with persistent GFP expression, without disturbing the tissue architecture, following a single administration of 4OH-tamoxifen to *GFRα1-CreER^{T2};CAG-CAT-EGFP* mice (Figures 2A and 2B; Figures S1A and S1B available online). After partial (~20%) pulse labeling of this population, long-term chase (months to over a year) revealed that labeled cells formed contiguous patches in which GFP+ cells were found in all stages of differentiation (Figure 2C). Importantly, the percentage of GFP+ cells out of the total GFRα1+ spermatogonia remained constant over the same extended period (Figure 2D). This indicates that the initially labeled GFRα1+ population (comprised of around 6,000 units = some 20% of the 30,000 total GFRα1+ units per testis) continually reproduced approximately the same number of GFRα1+ spermatogonia and gave rise to differentiating descendants that lost GFRα1 expression. This finding also shows that the influx of cells from outside the GFRα1+ compartment (i.e., Ngn3+, Kit+, or other unknown cells) is minimal in this undisturbed condition, consistent with previous reports (Nakagawa et al., 2007, 2010). To conclude, in steady state, the population of GFRα1+ spermatogonia function as the stem cells.

Intricate Clonal Fate Behavior of Pulse-Labeled Individual GFRα1+ Spermatogonia

Then we were motivated to investigate how individual GFRα1+ spermatogonia behave to achieve such population-level stem cell dynamics, using the same mouse model. With a lower dose of 4OH-tamoxifen, label was introduced into GFRα1+ cells sparsely (one labeled unit per ~6 mm tubule length on average), so that the fate of each "clone" (defined as a cohort of cells derived from a single labeled unit, regardless of whether or not they have split into multiple units) can be analyzed (Figure 2E). Over the time course of 2–20 days postinduction, the constituent units of individual clones were scored according to their GFRα1 expression and unit length by whole-mount immunostaining of seminiferous tubules. Shortly (2 days) after induction, the majority of labeled clones contained a single GFRα1+ unit (Figures 2F–2K). However, intriguingly, the subsequent clonal fates did not follow a regular pattern, but were highly variable between clones: for example, at day 14, some clones were observed to

(L and M) Average number of GFRα1+ and GFRα1− units (L) and cells (M) over the total clones. In (M), syncytia of 32 or more cells, all of which were GFRα1− and observed 4 or more days after the pulse, were scored as 32-cell syncytia because of the difficulty in making a precise count; this method underestimates the number of GFRα1− cells (broken line).

(N) Composition of the unit length of total pulse-labeled GFRα1+ spermatogonia over time, compared with steady-state tissue composition. Data in (L)–(N) show averages ± SEM (n = 3, 4, 5, 4, 4, 6, and 3 testes for 2, 4, 6, 10, 14, and 20 days postinduction, respectively). Scale bars, 50 μm throughout. The row data for (J)–(N) are shown in Table S1.

A

unit	total observed time (total unit number)	events	products	counts	ave. rate (event/day)
A_s	7982 h (171)	cell division	2xA_s	2	1/166
			A_{pr}	33	1/10
		death		7	1/48
A_{pr}	2779 h (70)	cell division	$A_{al\text{-}4}$	10	1/12
		fragmentation	2xA_s	5	1/23
		death		0	—
$A_{al\text{-}3}$	63 h (3)	n.d.			
$A_{al\text{-}4}$	1287 h (31)	cell division	$A_{al\text{-}8}$	4	1/13
		fragmentation	4xA_s	1	1/7
			2xA_s+A_{pr}	3	
			A_s+$A_{al\text{-}3}$	3	
			2xA_{pr}	1	
		death		0	—
$A_{al\text{-}8}$	72 h (4)	n.d.			

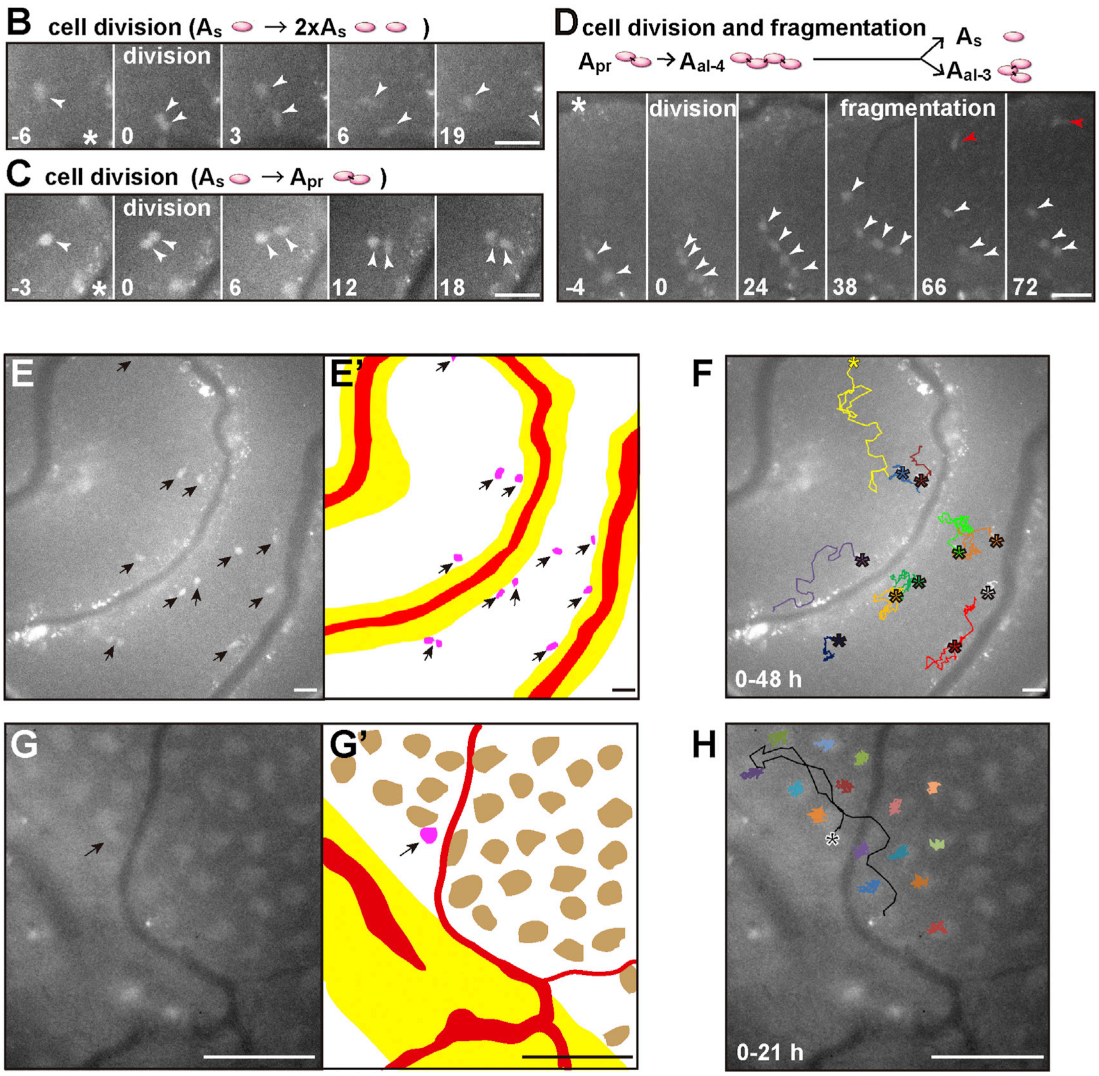

(legend on next page)

contain multiple GFRα1+ units only (where the GFRα1+ unit number increased without producing differentiation-destined GFRα1− progeny) (Figure 2G); some contained one GFRα1+ and multiple GFRα1− units (where the GFRα1+ unit number was maintained, while producing GFRα1− progeny) (Figure 2H); and others contained GFRα1− units only (where the GFRα1+ unit was lost altogether) (Figure 2I). The degree of clonal variation in the number of GFRα1+ and GFRα1− units (and cells) broadened progressively over time (Figures 2J, 2K, and S2; Table S1).

Despite the apparent variability in the individual clonal fates, the average of more than 100 clones at each time point recovered a conventional steady-state stem cell behavior through this period: in particular, the average number of GFRα1+ units per clone remained close to one, whereas the number of GFRα1− units (and, more dramatically, GFRα1− cells) per clone steadily increased (Figures 2L and 2M). Further, the composition of GFRα1+ units with different lengths across numerous clones remained largely constant over time, commensurate with that of the total GFRα1+ population observed by immunostaining of fixed samples (Figure 2N). Altogether, these findings are consistent with the dynamics of population asymmetry, in which maintenance of stem cells and production of differentiating descendants are balanced at the population level through continuous loss and replacement of stem cells (Klein and Simons, 2011).

Cell Division and Syncytial Fragmentation of GFRα1+ Spermatogonia Observed by Live Imaging

We then investigated the behavior of GFRα1+ spermatogonia by means of in vivo live imaging of *GFRα1-EGFP* knockin mouse testis (Uesaka et al., 2007) (Figure S1C), exploiting a procedure reported previously (Yoshida et al., 2007). Theoretically, the observed increase in cell number per pulse-labeled clone indicates the process of cell division (either complete or incomplete), whereas the increase in unit number per clone provides evidence of complete division ($A_s \rightarrow 2 \times A_s$) and/or syncytial fragmentation. Indeed, all of these expected processes were observed directly in the live imaging. Since continuous live imaging was feasible up to ~3 days, the average rates of these processes were measured by collecting data from multiple time courses (Figure 3A).

Unexpectedly, following a total of ~8,000 hr of observation, only two cases out of 35 divisions of GFRα1+ A_s cells were found to be complete, leading to the generation of two A_s spermatogonia (translating to a rate of once per 5–6 months), whereas the vast majority of divisions were incomplete and gave rise to one A_{pr} (Figures 3A–3C; Movies S1 and S2). Within a syncytium (A_{pr} and A_{al}), cell division was always incomplete and synchronous, leading to the doubling of syncytial length (e.g., $A_{pr} \rightarrow A_{al-4}$). Of particular note, fragmentations of GFRα1+ syncytia were observed at a frequency much higher than that of Ngn3+ syncytia (estimated at around once per 4 months per bridge) (Nakagawa et al., 2010) and even comparable to that of cell division (Figures 3A and 3D; Movie S3). This effectively replenishes the shorter units lost through incomplete divisions. Because of the half-life of the EGFP protein (2–3 days), the live-imaging study could not resolve their transition to Ngn3+ cells, based on their loss of EGFP fluorescence during the filming time available. However, the clonal fate of pulse-labeled GFRα1+ spermatogonia 2 days postinduction indicated that the GFRα1+→ Ngn3+ transition occurred in all categories of A_s, A_{pr}, and A_{al} GFRα1+ spermatogonia (Figure S3A), consistent with previous live imaging of *Ngn3-EGFP* mouse testes (Nakagawa et al., 2010). The death of GFRα1+ units was observed only rarely (Figure 3A).

Altogether, these observations indicate that GFRα1+ cells continually change their states between A_s, A_{pr}, and A_{al} spermatogonia through a combination of incomplete division and syncytial fragmentation, while giving rise to Ngn3+ cells from all of these states.

Rates of Incomplete Division and Syncytial Fragmentation of GFRα1+ Spermatogonia

In addition to these qualitative implications, the live-imaging study further provides quantitative insight into the dynamics of GFRα1+ cells. First, the rate of cell division (essentially incomplete) appeared to be independent of unit length because A_s, A_{pr}, and A_{al-4} syncytia all divide at around once per 10 days (Figure 3A). Second, the average fragmentation frequency of A_{pr} (one bridge) was around once per 20 days, whereas that of A_{al-4} (three bridges) was proportionately higher at around once per 7 days (Figure 3A), suggesting that each bridge breaks around once per 20 days, independent of unit length. Intriguingly, the fragmentation of A_{al-4} syncytia provided, instead of a regular pattern, fragments involving all possible permutations, viz. $4xA_s$, $2xA_{pr}$, $2xA_s+A_{pr}$, or A_s+A_{al-3} (Figure 3A), at frequencies compatible with stochastic breakdown of intercellular bridges once a syncytium is licensed for fragmentation (Figure S3B). Therefore, incomplete cell division and syncytial fragmentation of GFRα1+ spermatogonia appear to occur at constant rates, independent of the unit length.

Figure 3. Dynamics of GFRα1+ Spermatogonia Observed by Live Imaging

(A) Summary of cell division, fragmentation, and death of GFRα1+ spermatogonia observed in live imaging of *GFRα1-EGFP* knockin mouse testes. Average rates of each event are calculated as "counts of observed events"/"total observation time." nd, not detected.

(B–D) Examples of an $A_s \rightarrow 2 \times A_s$ division (B), an $A_s \rightarrow A_{pr}$ division (C), and an $A_{pr} \rightarrow A_{al-4}$ division, followed by a fragmentation into an A_s (red arrowhead) and an A_{al-3} (D), shown in selected frames of Movies S1, S2, and S3, respectively. Arrowheads, GFRα1-GFP+ spermatogonia; numerals, elapsed time relative to the cell division (hr). Asterisks, blood vessels.

(E and F) Localization and movement of GFRα1+ spermatogonia observed in live imaging. (E and E') A representative image of the surface of *GFRα1-EGFP* mouse testis (the first frame of Movie S4) is shown. (E') Trace of (E) showing GFRα1+ spermatogonia (magenta), blood vessels (red), and interstitium (yellow). (F) Trajectories of individual GFRα1-GFP+ spermatogonia over 48 hr of observation (Movie S4), shown in different colors.

(G and H) Movement of GFRα1+ spermatogonia among the immobile Sertoli cells. (G) The first frame of the live imaging of *GFRα1-EGFP; GATA1-EGFP* mouse testis (Movie S5). (G′) Trace of (G) showing a GFRα1+ spermatogonium (magenta), Sertoli cells (brown), blood vessels (red), and interstitium (yellow). (H) Trajectories of a GFRα1-EGFP+ spermatogonium (black line) and GATA1-EGFP+ Sertoli cells (colored lines) during 21 hr of observation, overlaid on the first frame. Asterisks indicate the starting positions; scale bars, 30 μm.

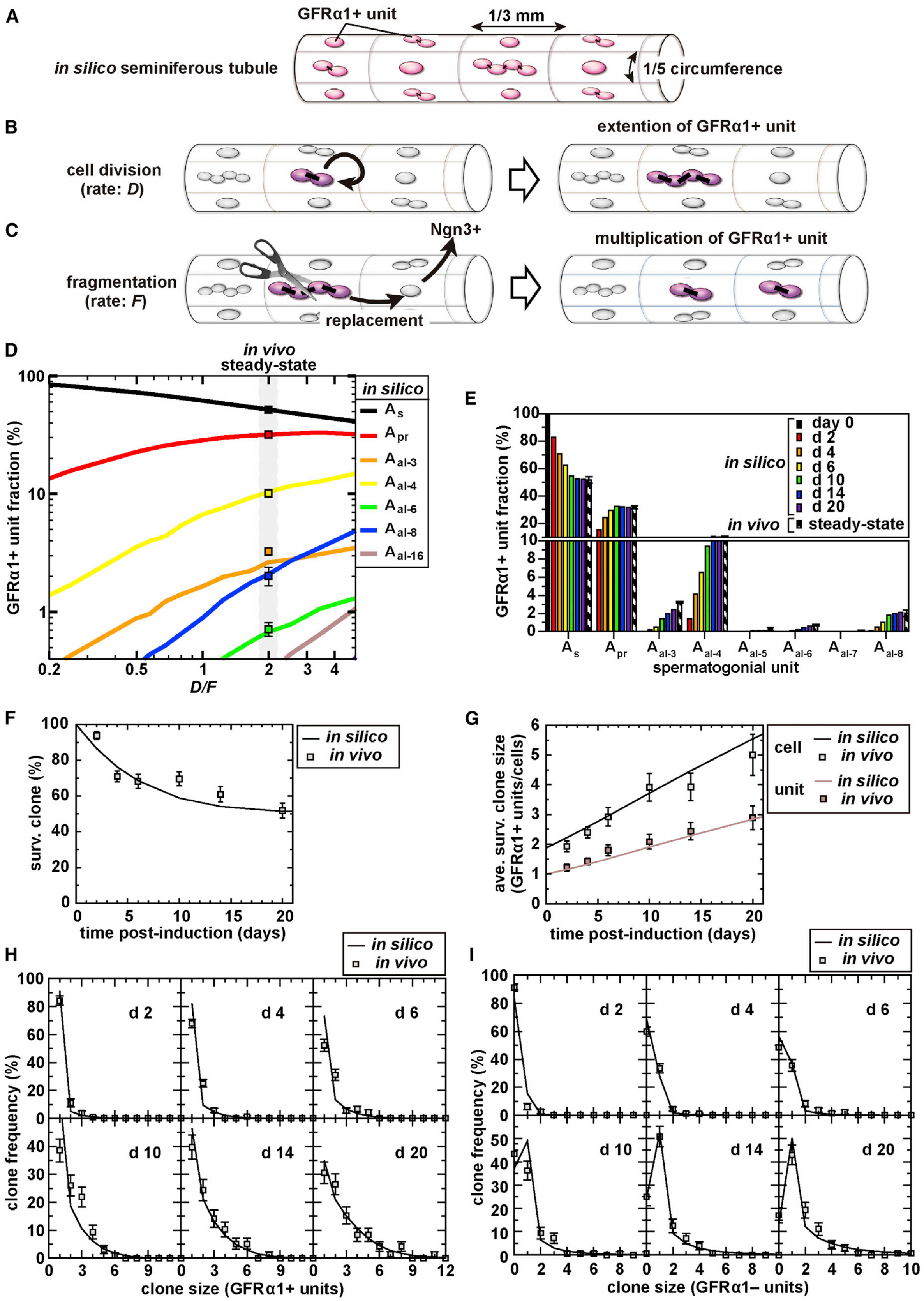

(legend on next page)

Active Movement of GFRα1+ Spermatogonia around the Vasculature-Associated Region

In common with the entire population of undifferentiated spermatogonia (Chiarini-Garcia et al., 2001; Yoshida et al., 2007), the GFRα1+ subpopulation tends to localize near the vasculature and interstitium surrounding the tubules (Figures S3C, S3C′, and S3F–S3G′). Moreover, from the live-imaging study, it was apparent that GFRα1+ spermatogonia were in constant movement in the basal compartment (Figures 3E, 3E′, and 3F; Movie S4). Whereas the majority preferentially moved within the vasculature-proximal region, others migrated from one such region to another. Without showing any systematic pattern, cells were seen to actively weave their way through the ordered network of immobile Sertoli cells (Figures 3G, 3G′, and 3H; Movie S5), over a range of approximately 20–150 μm within a single day. This contrasts with the behavior of Ngn3+ spermatogonia, which are less motile in the vasculature-associated region, before actively spreading over the basal compartment on transition into A_1 spermatogonia (Yoshida et al., 2007).

Synthesis of a Minimal Biophysical Modeling Scheme

Considering the aforementioned observations of highly variable clonal fates, continual conversion between the states of A_s, A_{pr}, and A_{al} and active movement in the tissue, the behavior of GFRα1+ spermatogonia may seem unconstrained. However, the observation that incomplete division and syncytial fragmentation occur at constant rates, independent of unit length, may suggest simple rules underlying such complex behaviors. We were motivated, therefore, to try to capture the dynamics of GFRα1+ spermatogonia using a biophysical modeling scheme. In particular, we synthesized a model relying solely on parameters inferred from live imaging (the foregoing two rates), as well as their density and localization in seminiferous tubules. Then the validity of the model was evaluated by testing whether it was able to predict the wide range of independent data, including the intricate clonal fates revealed by the pulse-labeling study. Here, the model was designed to be as simple as possible, with minimal parameters, so that we could capture the basic principles of the dynamics of GFRα1+ spermatogonia, as described below. For further details of the modeling scheme, see the Supplemental Experimental Procedures.

As seen above, although the GFRα1 units are scattered and moving around the tissue, their local density (viz. pool size) is maintained largely constant. In formulating the model, we aimed to capture the dynamics of GFRα1+ units under the condition of such a constant density. To reflect this, we considered a modeling scheme in which the basal compartment of the seminiferous tubules was divided into domains that accommodate one GFRα1+ unit each (Figure 4A). Based on their measured average density (17 units/mm tubule) and affinity to the vasculature (Figures S3C, S3C′, and S3F–S3G′), whose average number around a tubule is 5.2 (Klein et al., 2010), we divided the circumference into five domains of 1/3 mm in length (Figure 4A). In this scheme, each domain corresponds to the approximate territory of a single GFRα1+ unit.

Then to reflect our live-imaging observations, all the constituent GFRα1+ spermatogonial units were allowed stochastically to undergo incomplete cell division that doubles the unit length, at a constant rate (defined as *D*), independent of unit length (Figure 4B). In addition, intercellular bridges were allowed to break stochastically, leading to the fragmentation of a syncytium into multiple units, at a constant rate (defined as *F*) per bridge throughout all the GFRα1+ syncytia (Figure 4C). The pattern of fragmentation was set to occur randomly among the bridges (with any bridge breaking with a probability of 50%), consistent with observation (Figure S3B). To achieve a constant density of GFRα1+ units, the genesis of a new GFRα1+ unit by fragmentation was set to accompany the GFRα1+ → Ngn3+ transition in any one of the neighboring domains and the replacement of the lost GFRα1+ unit by the newly formed GFRα1+ unit (Figure 4C). Such translocation between domains was consistent with the observed movements of GFRα1+ cells (Figures 3E–3H).

To reduce the complexity of the model, we did not include the infrequent process of $A_s \rightarrow 2 \times A_s$ complete division (Figures 3A and 3B), which is in any case implicit in the sequential occurrence of an $A_s \rightarrow A_{pr}$ division, followed by $A_{pr} \rightarrow 2 \times A_s$ fragmentation. Nor did we allow for the death of GFRα1+ spermatogonia or Ngn3+ → GFRα1+ reversion, reflecting the low frequency of these processes (Figure 3A) (Nakagawa et al., 2010). Reflecting their low fragmentation frequency, we could assume that GFRα1− (Ngn3+ and more advanced) units do not fragment but simply accumulate at each lattice site. Finally, GFRα1− units were also set to follow a low rate of death (once per 30 days), consistent with observation (Huckins and Oakberg, 1978). However, within the framework of the model, death of GFRα1− units does not, in any case, affect the dynamics of the GFRα1+ population.

Figure 4. Model Prediction of the In Vivo Dynamics of GFRα1+ Spermatogonia

(A) An imaginary seminiferous tubule used as the framework for the modeling scheme: the basal compartment is modeled as a regular cylindrical lattice, in which each domain accommodates one GFRα1+ unit.

(B and C) Elementary processes introduced into the model. With the rate of "*D*," a GFRα1+ spermatogonial unit divides incompletely to double its length (B). With the rate of "*F*" per bridge, a GFRα1+ syncytium fragments into multiple pieces; this event is allied with the GFRα1+ → Ngn3+ transition of neighboring unit(s).. As a result, newly generated units replace neighboring units and persist as GFRα1+ (C). For details, see the main text and the Supplemental Experimental Procedures.

(D) Dependence of the steady-state unit composition on the ratio *D*/*F* predicted in silico (multicolored lines), in which the rates measured from live imaging (*D* = once/10 days; *F* = once/20 days/bridge; *D*/*F* = 2.0) captured the in vivo steady-state composition obtained from whole-mount immunostaining (squares).

(E) Convergence in silico to steady-state composition of GFRα1+ units from an initial condition in which all GFRα1+ units are A_s, using the rate constants *D* = once/10 days and *F* = once/20 days/bridge.

(F–I) Model prediction captures clonal fate behaviors of GFRα1+ units observed in vivo over the 20 day time course, represented by a percentage of surviving clones out of total clones (F), average number of GFRα1+ units(cells) in individual surviving clones (G), and clone size distribution for GFRα1+ (H) and GFRα1− (I) units. Throughout, lines show the in silico predictions using the same *D* and *F* rates, whereas the experimental data are shown by squares (average ± SEM among testes). (H) and (I) are replotted from Figures 2J and 2K.

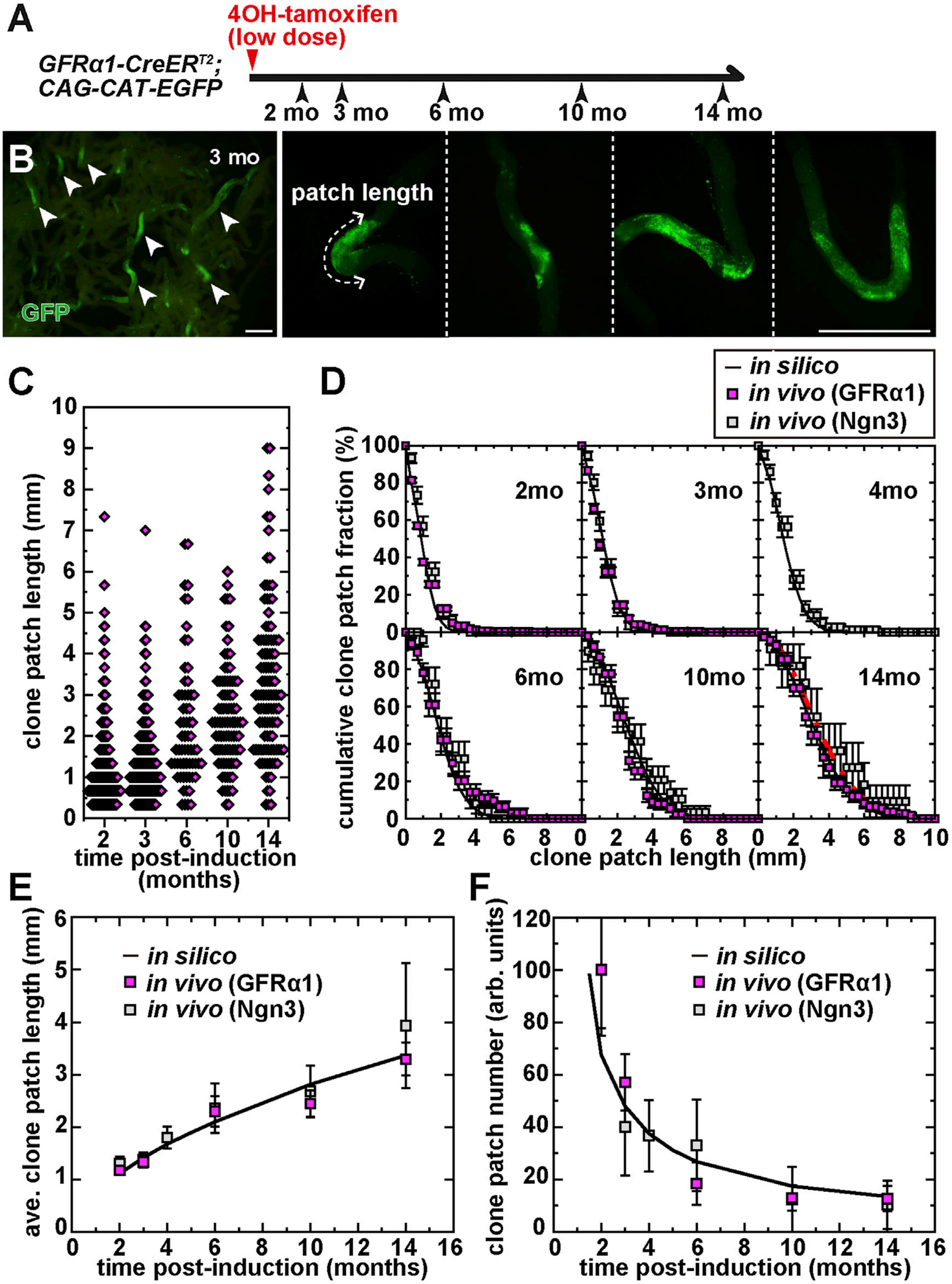

Figure 5. Long-Term Dynamics of GFRα1+ Spermatogonia-Derived Clones

(A) Experimental schedule for the long-term clonal analysis of pulse-labeled GFRα1+ cells.

(B) Seminiferous tubules at 3 months postlabeling, showing GFP+ clonal patches (arrowheads) and their higher magnifications with measurement of the patch length. Scale bars, 1 mm.

(C) Distribution of clonal patch lengths at 2, 3, 6, 10, and 14 months postinduction.

(legend continued on next page)

Model Prediction of the In Vivo Dynamics of GFRα1+ Spermatogonia

We then questioned whether the model had the capacity to predict the wide and complex range of independent in vivo measurements. In this scheme, the in silico dynamics of GFRα1+ spermatogonia is fully specified by just two parameters: the rates of cell division (*D*) and fragmentation (*F*). First, within the framework of the model, the GFRα1+ population is predicted to converge to steady state, in which GFRα1+ units acquire a particular composition that is independent of the initial condition but depends uniquely on the ratio *D*/*F* (colored lines in Figure 4D). Intriguingly, using the rates of *D* (= once per 10 days) and *F* (= once per 20 days per bridge) inferred from live imaging, the model faithfully recapitulated the steady-state composition of GFRα1+ spermatogonia measured in vivo (Figure 4D). The steady state is recovered rapidly (largely within 10 days, corresponding to one round of cell division on average), even from such an extreme initial condition in which all GFRα1+ units are A_s (Figure 4E). In addition to the proportions of GFRα1+ A_s, A_{pr}, and $A_{al\text{-}4}$ spermatogonia, the model correctly predicted the near-absence of GFRα1+ units larger than eight, as well as the small number of "odd-sized" units ($A_{al\text{-}3}$, $A_{al\text{-}5}$, $A_{al\text{-}6}$, and $A_{al\text{-}7}$), which was already a nontrivial test of the validity of the modeling scheme.

Second, we evaluated the extent to which the model can predict the detailed clonal fate dynamics of GFRα1+ units scored in the pulse-labeling experiment over 20 days postinduction (Figures 2E–2N; Figure S2; Table S1), with the same rates of *D* and *F*. In common with the in vivo observation (Figures 2J, 2K, and S2), in silico clones also followed variable fates. In the model, once the GFRα1+ → Ngn3+ transition occurs in all the units of a clone, such a clone never returns to the GFRα1+ compartment. As a result, the fraction of clones that retained at least one GFRα1+ unit (surviving rate) progressively diminishes over time (line in Figure 4F), which quantitatively recapitulated the in vivo measurements (squares in Figure 4F). As a consequence of the steady-state dynamics, it was also predicted that the average number of GFRα1+ units (and cells) of the "surviving" clones progressively increase, so that the average number of GFRα1+ units across all clones remains close to one. Indeed, these predictions quantitatively captured the in vivo observations (Figures 2L, 4G, and S4A). More significantly, the model prediction showed excellent agreement with the in vivo distribution of GFRα1+ units (and cells) at all data points over the time course of 20 days (Figures 2J, 4H, S2, and S4B). Finally, the average and distribution of the number of GFRα1− units per clone (i.e., units that had exited GFRα1+ compartment and transited to Ngn3+ and then more advanced spermatogonia) were also accurately predicted by the model (Figures 2K, 2L, 4I, and S4A).

To summarize, from an in silico modeling scheme that was synthesized solely from the local density and distribution of GFRα1+ units in seminiferous tubules and the rates of cell division and syncytial fragmentation inferred from the live-imaging study, we were able to accurately predict both the steady-state tissue composition of spermatogonial units (an independent measurement) and the intricate fate behavior of spermatogonial units (obtained from a totally independent pulse-labeling study). These findings provide strong support for the validity of the simple modeling scheme in capturing the in vivo steady-state behavior of GFRα1+ population, at least over the 20 day time course.

Long-Term Dynamics of GFRα1+ Population

We then assessed the capacity of the same model to predict the long-term (over months) clonal behavior in steady state. On this timescale, clones derived from the pulse-labeled single GFRα1+ units evolved into large (sometimes fragmented) patches in which the labeled GFRα1+ cells were overwhelmed in number by differentiating labeled cells. Because a count of unit(cell) number is unfeasible in this phase, we characterized clone size by the patch length along the axis of the tubule (Figures 5A and 5B). In common with the number of GFRα1+ units(cells) in each clone seen in the short-term pulse-labeling study up to 20 days, at 2, 3, 6, 10, and 14 months postinduction, the patch length showed a variable size distribution, with the average length increasing over time (Figures 5C–5E). In parallel, the number of surviving patches per testis decreased (Figure 5F). Significantly, extrapolation of the in silico dynamics to these longer times correctly predicted the average and distribution of patch length and surviving rate of GFRα1+ unit-derived clones, using the same rates of cell division and fragmentation, *D* (= once per 10 days) and *F* (= once per 20 days per bridge) (Figures 5D–5F).

In previous studies, pulse labeling of Ngn3+ spermatogonia was used to trace the fate of surviving stem cell clones over a 14 month time course (Klein et al., 2010; Nakagawa et al., 2007). Because the majority of Ngn3+ cells are destined for differentiation, these studies relied on the premise that the few Ngn3+ spermatogonia that had transited back into the GFRα1+ compartment behaved without distinction from the "innate" GFRα1+ cells (Nakagawa et al., 2010). This premise is strongly supported by the observation that the average and distribution of patch lengths as well as the rate of surviving clones are all consistent between GFRα1+ and Ngn3+ cell-derived clones and predicted by the same model at these long times (Figures 5D–5F).

To summarize, the minimal biophysical model, which was synthesized from the very short-term observations in live imaging (up to 3 days), is capable of capturing the fate behavior of GFRα1+ spermatogonia from the short term (up to 20 days) to the long term (up to times comparable with the life span of mice).

Dynamics of GFRα1+ Spermatogonia Following Tissue Insult

We then turned to investigate the dynamics of GFRα1+ spermatogonia following a strong perturbation from steady state.

(D) Comparison of clonal patch length distribution between in silico prediction (solid lines) and in vivo measurement (squares) over 14 months. Red dotted line in the panel of 14 months shows the scaling function obtained by Klein et al., 2010.

(E and F) Comparisons of the evolution of average patch length (E) and patch number per testis presented in arbitrary units (F) between in silico prediction (solid line) and in vivo measurements (squares). In (D), (E), and (F), magenta and gray squares indicate patches originated from GFRα1+ (replotted from C) and Ngn3+ units (replotted from Klein et al., 2010 and Nakagawa et al., 2007), respectively. Values are shown as average ± SEM.

To do so, we analyzed a partial germ cell depletion model induced by a moderate dose of busulfan (10 mg/kg). In this condition, testis shows acute and massive germ cell death, causing the reduction of the number of GFRα1+ units to a minimum of around one-third of the steady-state value by postinsult day 10. Then the number of GFRα1+ spermatogonia gradually comes back to their preinsult level in about 2 months (Nakagawa et al., 2010).

Experimentally, we pulse labeled the GFRα1+ cells on postinsult day 10 and analyzed their clonal fate in the following recovery phase (Figure 6A). Although the rate of Ngn3+ → GFRα1+ reversion increases substantially following insult (Nakagawa et al., 2010), the appearance of GFRα1+ cells through this process was limited during the observed period (Figure S5A). As with steady state, in regeneration, the size of individual pulse-labeled clones diverged over time in the number of both constituent GFRα1+ and GFRα1− units (Figures 6B and 6C). Interestingly, a significant portion of clones lost all GFRα1+ progeny even in regeneration. However, in contrast to steady state, the total number of GFRα1+ units (viz. the average number of GFRα1+ units per clone) increases because of a tilt in the overall balance of production of GFRα1+ and GFRα1− units toward the former (Figure 6D).

Then we questioned whether the same biophysical model could capture the clonal dynamics in regeneration, after seeding the lattice with GFRα1+ units in proportion to their observed density and syncytial composition. As the simplest adaptation of the model, we allowed syncytial fragmentation to be uncompensated by loss of a neighboring GFRα1+ unit, when the fragment migrates into a vacant domain. Under these conditions, we found that the predictions of the model showed a remarkable agreement with the measured clone dynamics, including the clone survival rate (Figure 6E), and the average and distribution of clone sizes (for both GFRα1+ units[cells] and GFRα1− units) (Figures 6D, 6F–6H, and S5B), over the wide range of time points for 18 days, if we made a minimal adjustment of the rate parameters. In particular, we found an optimal fit of the model to the data when the rates of cell division and fragmentation were both increased to around once per 8 days (from once per 10 days in steady state) and once per 10 days per bridge (from once per 20 days), respectively, while introducing a significant decrease of the death rate of GFRα1− cells (once per 160 days, from once per 30 days). Using these parameters optimized from the short-term clonal behavior (up to 18 days), the model also accurately predicted the recovery in the tissue density of GFRα1+ units and cells up to 2 months following insult, when the regeneration process largely completed (Figure S5C).

These results provide further support for the general validity and predictive power of the modeling scheme. Moreover, these results suggest that the dynamics of GFRα1+ cells in regeneration following tissue injury is not based on a distinct program but follows the same pattern of stochastic rules as that seen in steady state.

DISCUSSION

Motivated by recent observations that question the validity of the prevailing "A_s model," this study explored an alternative theory of the identity and behavior of mouse spermatogenic stem cells and conducted single-cell-resolution analyses of the behavior of GFRα1+ spermatogonia. The live-imaging study revealed that, in the GFRα1+ compartment, practically all cell divisions are incomplete, whereas syncytial fragmentation occurs rather frequently, and that these processes follow constant rates that are independent of unit length. Based on these two measured rates, as well as cell density and localization in seminiferous tubules, we developed a minimal biophysical model to describe the dynamics of GFRα1+ spermatogonia. This model could predict the range of complex data obtained from independent measurements, from the steady-state composition of GFRα1+ units to the wide range of intricate clonal fate behaviors of pulse-labeled GFRα1+ cells, both in steady state and in regeneration. Given the ability of such a highly simplified model to predict the complex in vivo behavior, we concluded that the principles that define the dynamics of GFRα1+ compartment have been successfully resolved. On the other hand, the contribution of rare events, such as cell death or possible deviation from stochasticity, should be small enough to capture the overall dynamics of stem cells, although these factors should affect the detailed behavior of stem cells.

Figure 7A illustrates the stem cell dynamics proposed in this study, in which GFRα1+ units continuously extend via incomplete division and fragment via intercellular bridge breakdown, while giving rise to Ngn3+ progeny. In this scheme, individual GFRα1+ cells constantly change their state reversibly between single cells and variable lengths of syncytia; Figure 7B represents a typical example of such stochastic fate behavior predicted in silico. Yet, through this process at the population level, the number and composition of GFRα1+ cells, as well as the production rate of Ngn3+ cells, were kept constant. Therefore, we propose that the entirety of GFRα1+ spermatogonia comprises a single "stem cell pool."

Onset of Ngn3 expression represents the exit from the stem cell compartment toward differentiation. Although this transition does not indicate the loss of reversibility, Ngn3+ cells show a pronounced differentiation bias. Indeed, in steady state, the vast majority of pulse-labeled Ngn3+ cells differentiate into spermatozoa and disappear from the tissue after a couple of months. When cells become Kit+, they appear to further decrease in potential to return into the GFRα1+ compartment (Figure 7A, black broken arrows) (Barroca et al., 2009; Nakagawa et al., 2007, 2010). In parallel, the frequency of (incomplete) cell division increases, whereas syncytial fragmentation becomes more infrequent (Huckins and Oakberg, 1978; Nakagawa et al., 2010). As a result, the bulk of Ngn3+ and Kit+ spermatogonia extend their unit length unidirectionally as differentiation progresses (Figure 7A).

In mouse spermatogenesis, syncytial fragmentation was proposed based on the odd-numbered spermatogonial units observed after irradiation (Erickson, 1981) and directly filmed for the first time in Ngn3+ spermatogonia (Nakagawa et al., 2010). Given its low frequency in steady state, this process has been considered functionally significant in regeneration, where fragmentation appears to occur much more frequently (Nakagawa et al., 2010; Spradling and Fan, 2010). However, the current study revealed that, in the GFRα1+ compartment, syncytial fragmentation regularly occurs in steady state at a frequency comparable with that of cell division and that this

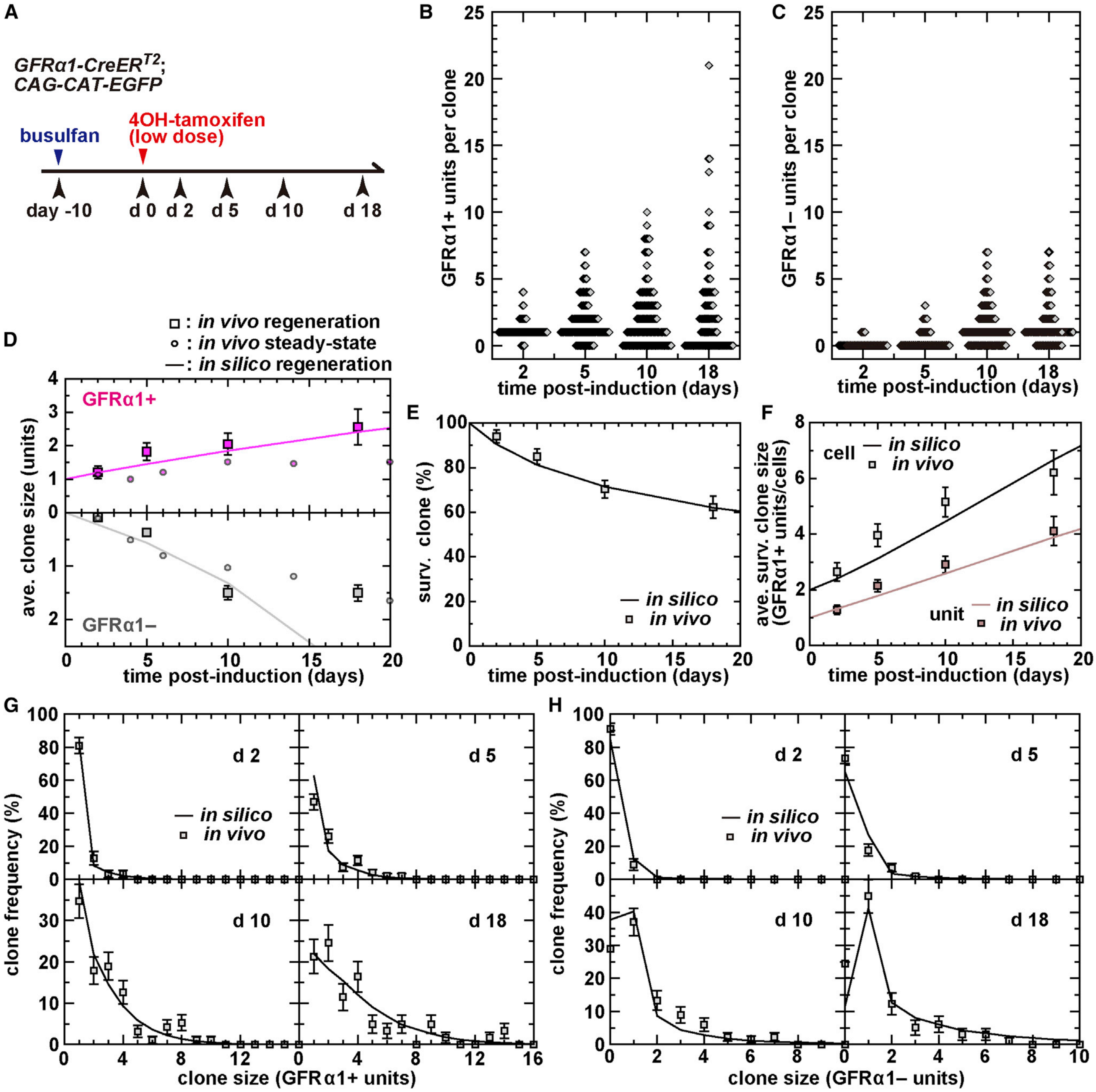

Figure 6. Dynamics of GFRα1+ Spermatogonia during Regeneration

(A) Experimental schedule for clonal analysis of GFRα1+ spermatogonia during regeneration.

(B and C) Distribution of clone size over 18 days after induction scored by the number of GFRα1+ (B) and GFRα1− (C) units.

(D–H) The observed in vivo clonal fate behavior of the pulse-labeled GFRα1+ units in regeneration (squares; shown as average ± SEM), and their recapitulation by in silico model prediction after fitting for the rates of *D* and *F* and death of GFRα1− units (solid lines; see main text): average numbers of GFRα1+ (upper) and GFRα1− (lower) units per clone compared with those in steady-state (small circles; reproduced from Figure 2L) (D), the percentage of surviving clones (E), the average number of GFRα1+ unit(cell) per clone (F), and the clone size distribution scored by the number of GFRα1+ (G) and GFRα1− units (H) (replotted from B and C, respectively).

process plays a fundamental role in the maintenance of stem cell pool and the continuity of spermatogenesis.

Within the modeling scheme established in this study, the maintenance of the GFRα1+ population relies on the balance between their multiplication (syncytial fragmentation) and loss (transition into Ngn3+), which are locally coordinated. This provides a cell-level explanation for the population asymmetry of mouse spermatogenic stem cells that was first discovered by large-scale (millimeters of patch length) and long-term (over months of chase period) clonal fate analysis of pulse-labeled

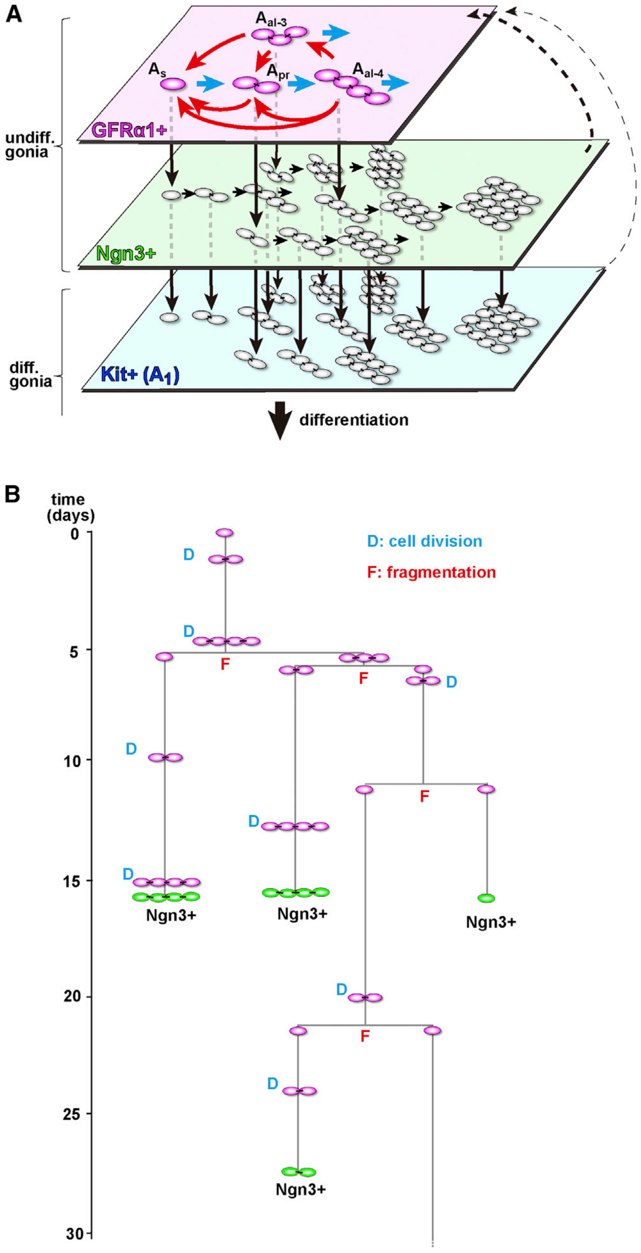

Figure 7. A Proposed Stem Cell Dynamics of Mouse Spermatogenesis

(A) A scheme of the proposed stem cell dynamics. On the top of the differentiation hierarchy, GFRα1+ spermatogonia comprise a single stem cell pool, in which cells continually and reversibly interconvert between states of A_s, A_{pr}, and A_{al} spermatogonia through incomplete cell division (blue arrows) and syncytial fragmentation (red arrows), while giving rise to Ngn3+ cells. After leaving the GFRα1+ compartment, differentiation-destined cells follow a series of transition (GFRα1+ → Ngn3+ → Kit+; downward black arrows) that accompanies the extension of syncytial length (rightward black arrows). Ngn3+ and, to a lesser extent, Kit+ cells retain the capacity to revert into the GFRα1+ compartment in a context-dependent fashion (broken arrows).

(B) Pedigree of a GFRα1+ unit-derived clone evolved in the in silico modeling scheme, representing a typical interconversion between A_s and syncytial states through incomplete cell division (D) and fragmentation (F), as well as generation of Ngn3+ spermatogonia.

Ngn3+ cells (Klein et al., 2010; Nakagawa et al., 2007). Supporting this idea, the rate of stem cell loss and replacement and the long-term scaling property of the clonal patch length distribution obtained by Klein et al. (2010) agree quantitatively with those predicted by the current biophysical model (Figure 5D; Supplemental Experimental Procedures).

In tissues without formation of syncytia, population asymmetry is typically achieved by local correlation of the division and loss (differentiation) of stem cells (Klein and Simons, 2011). In mouse spermatogenesis, the striking capacity of the model to capture the clonal fate behavior suggests that syncytial fragmentation may be linked with loss (transition to Ngn3+). However, the current study can address neither the causal relationship between fragmentation and loss nor the mechanism that coordinates these processes. To answer these questions, it is important to reveal the (yet unknown) mechanism that keeps the local density of GFRα1+ units constant in seminiferous tubules, a feature that was built into the construction of our current modeling scheme.

This study highlights a long-held question in germ cell research: what is the biological significance of intercellular connection? After meiosis, it is established that the connection ensures the formation of equivalent gametes from haploid spermatids, utilizing the shared cytoplasmic gene products, including those from X and Y chromosomes (Braun et al., 1989). However, the role of the connection remains an open question for diploid spermatogonia. Given the theory of equipotent stem cell pool composed of the entire GFRα1+ spermatogonia, the connection appears to be unrelated to their stem cell potential. However, Ngn3+ and Kit+ spermatogonia harbor more stable intercellular bridges, suggestive of some unknown role of intercellular connection in this differentiation-destined compartment. Further investigations are warranted to address this fundamental question.

Another important finding of this study is that the seemingly complex dynamics of the GFRα1+ compartment can be effectively described only by the rates of incomplete cell division (*D*) and syncytial fragmentation (*F*), the governing parameters of the modeling scheme. This notion is strengthened by the analysis of regeneration (Figure 6), which suggests that these rates are regulated in parallel throughout the GFRα1+ compartment, independent of their unit length, so that GFRα1+ spermatogonia can rapidly recover their pool size. Interestingly, the steady-state composition of GFRα1+ units with different lengths sensitively reflects the ratio of these rates, *D/F* (Figure 4D). In this scheme, the generation and frequency of spermatogonial units are simply a reflection of this ratio, including not only A_s, A_{pr}, A_{al-4}, A_{al-8}, etc., that are considered "regular" in the "A_s model" but also those with 3, 5, 6, 7 cells, etc., that are often considered "irregular." Together with the mutual interchange of these morphologically different states, we propose that all GFRα1+ units are equally regular and, in particular, that A_s is not a special entity. We also propose here the nomenclature of A_{al-3}, A_{al-5}, A_{al-6}, A_{al-7}, etc., without distinction from A_s, A_{pr}, A_{al-4}, A_{al-8}, etc.

Although the results of this study fully support the theory of a single stem cell pool comprised of functionally equivalent A_s and syncytial GFRα1+ spermatogonia (Figure 7A), one may also conjecture that a small population of slow-cycling GFRα1+ A_s cells that always undergo complete division act as

the "true" stem cells. If this were the case, then one would expect a transfer of the clonal dynamics from that represented by the behavior of short-lived cells (which interconvert between A_s and syncytia) to that of the slow-cycling A_s population over a long time course. However, such transfer was not observed in the fate behavior of GFRα1+ cells over extended timescales, from days to over a year. Therefore, although we do not rule out the presence of such a slow-cycling compartment, we can conclude that their contribution (would they exist) is not essential to maintain life-long spermatogenesis in mouse (for details, see the Supplemental Experimental Procedures). It remains open, however, in other animals with longer longevities, whether the same scheme of a single stem cell pool can be extrapolated to the years- or decades-long spermatogenesis or whether some slow-cycling population plays a significant role. Interestingly, primate testes host a large number of immature spermatogonia, A_{dark}, which appear not to have rodent counterparts (Hermann et al., 2010). Elucidating the roles of this population would be warranted to address this interesting question.

In this study, the live-imaging observation also revealed a unique property of GFRα1+ spermatogonia: the active movement over the seminiferous tubules. To orchestrate the local coupling of syncytial fragmentation and loss (transition into Ngn3+) of sparsely distributed GFRα1+ spermatogonia, it is vital that GFRα1+ cells are able to freely relocate. Failure to do so would elicit progressive unevenness in the local density of GFRα1+ cells, which would eventually compromise the integrity of the tissue. Indeed, the high motility of stem cells observed in mouse spermatogenesis may be paradigmatic of systems in which the stem cell niche is "facultative" or "open" (Morrison and Spradling, 2008; Stine and Matunis, 2013). This shows a stark contrast with other systems supported by "definitive" or "closed" niche, such as *Drosophila* germline, mammalian intestinal crypt, or hair follicles, where stem cells lie in close association with each other and remain attached to a localized niche structure, and their movement must be limited within the niche region (Fuller and Spradling, 2007; Lin and Spradling, 1997; Sheng and Matunis, 2011). In future studies, it will be important to understand how the movement of stem cells is controlled and regulated by the interaction with such niche environments.

Finally, although incomplete division and syncytial fragmentation are germ cell specific, this study may provide important insights for other stem-cell-supported systems. In particular, we show that stem cells can be defined, not as a particular cell type, but as a heterogeneous population in which cells continually interconvert between different states. Indeed, such "dynamical heterogeneity" resonates with the recent live-imaging study of hair follicle stem cells, which show that self-renewal potential may be correlated with position within the stem cell niche (Rompolas et al., 2013).

EXPERIMENTAL PROCEDURES

Animals

GFRα1^{CreERT2}knockin mice were generated as described in Figure S1A. *GFRα1*^{EGFP} (Uesaka et al., 2007), *CAG-CAT-EGFP* (Kawamoto et al., 2000), and *GATA1-EGFP* (Suzuki et al., 2009b) alleles were as previously described. All the mice used in this study were heterozygous for one or two of these alleles and simply indicated by their allelic name(s), with the background of C57BL/6 (from Japan CLEA and Japan SLC and used as wild-type animals). Although mice carrying one nonfunctional knockin allele of the *GFRα1* gene, *GFRα1*^{CreERT2}, and *GFRα1*^{EGFP} were used for pulse-labeling and live-imaging experiments, respectively, such heterozygosity neither affected the total density and composition of GFRα1+ units nor the overall integrity of spermatogenesis, over 1 year postlabeling (Figures S1D–S1I). All animal experiments were conducted with approval of The Institutional Animal Care and Use Committee of National Institutes of Natural Sciences, unless specifically mentioned.

Whole-Mount Immunofluorescence of Seminiferous Tubules

Immunostaining of whole-mount seminiferous tubules was performed as previously described (Nakagawa et al., 2010) using anti-GFRα1 Ab (1:1,000 dilution; R&D Systems), anti-GFP Ab (1:300 dilution; Invitrogen), and anti-Kit Ab (1:200 dilution; BD Biosciences). Observation and photography were performed with a BX51 upright fluorescence microscope equipped with a DP72 CCD camera (Olympus). Spermatogonia were judged as belonging to a syncytium when, based on a continuous GFRα1 or GFP staining using a 60× water immersion objective lens, the cell-cell connection was visually detected. To measure the lengths of the patches of GFP+ cells, M205C stereomicroscope with a DFC490 CCD camera (Leica) was used.

Pulse Labeling of GFRα1+ Spermatogonia

Three-month-old *GFRα1-CreER*^{T2}*; CAG-CAT-EGFP* mice were injected intraperitoneally with 2.0 mg (for a higher labeling efficiency) or 0.25–0.35 mg (for clonal labeling) per individual of 4OH-tamoxifen (Sigma) dissolved in ethanol, in dimethyl sulfoxide, and then in sesame oil (Nakalai Tesque). For clonal lineage tracing, their testes were processed for whole-mount immunofluorescence. To induce regeneration, mice were intraperitoneally injected with busulfan (10 mg/kg) as described previously (Nakagawa et al., 2010) prior to 4OH-tamoxifen administration.

Intravital Live Imaging

Live-imaging of the testes of 4- to 5-month-old *GFRα1*^{EGFP} or *GFRα1*^{EGFP}*; GATA1-EGFP* mice under anesthesia was performed as described before, using epifluorescence IX61WI microscope (Olympus) (Yoshida et al., 2007). Time-lapse images were captured at the rate of one flame per 30 min using the Andor iXon EM-CCD camera controlled by Metamorph software (Molecular Devices). Movies were constructed by Metamorph software, and the trajectories of spermatogonia and Sertoli cells were manually extracted from movies using Metamorph and ImageJ software. An intercellular bridge was deemed to be intact if the cells remained within 30 μm for more than 12 hr (Supplemental Experimental Procedures). Although only GFRα1+ cells located in the superficial region of the testis were filmed, their representativeness is supported by the agreement of the densities and compositions of GFRα1+ units measured by live-imaging and whole-mount immunostaining studies (the latter represents the average values of the entire tubules) (Figures S3C–S3E). It is further consolidated by the correspondence between rates of cell division and syncytial fragmentation measured from live-imaging data and the range of fate behavior of pulse-labeled GFRα1+ cells found evenly throughout the testis (see text).

SUPPLEMENTAL INFORMATION

Supplemental Information for this article includes Supplemental Experimental Procedures, five figures, one table, and five movies and can be found with this article online at http://dx.doi.org/10.1016/j.stem.2014.01.019.

AUTHOR CONTRIBUTIONS

K.H., B.D.S., and S.Y. designed the experiments, performed data analysis, and wrote the manuscript. K.H. and T.N. performed in vivo experiments. B.D.S. performed in silico analyses. H.E., M.S., and M.Y. generated genetically modified animals.

ACKNOWLEDGMENTS

We thank J. Milbrandt for providing *GFRα1-EGFP* and *GFRα1-CreER*^{T2} mice and J.-I. Miyazaki for providing *CAG-CAT-EGFP* mice. We are grateful to

R. Sugimoto and K. Inada for technical instruction and assistance. We are grateful to K. Hara-Harikae, K. Ikami, Y. Kitadate, A.M. Klein, H. Mizuguchi, T. Nagasawa, Y. Nakamura, T. Ogawa, A. Philpott, M. Tokue, and E. Watanabe for useful discussions and encouragement and Y. Kuboki for secretarial assistance. We thank members of the Model Animal Research Facility of the National Institute for Basic Biology Bioresource Center for animal care. This work was partly supported by a Grant-in-Aid for Scientific Research (KAKENHI; 20116004, 24247041, and 25114004 to S.Y. and 22780267 to K.H.), Wellcome Trust (098357/Z/12/Z to B.D.S.), and the National Institutes of Natural Sciences International Exchange Program (to S.Y. and K.H.).

Received: October 7, 2013
Revised: December 20, 2013
Accepted: January 29, 2014
Published: May 1, 2014

REFERENCES

Barroca, V., Lassalle, B., Coureuil, M., Louis, J.P., Le Page, F., Testart, J., Allemand, I., Riou, L., and Fouchet, P. (2009). Mouse differentiating spermatogonia can generate germinal stem cells in vivo. Nat. Cell Biol. *11*, 190–196.

Braun, R.E., Behringer, R.R., Peschon, J.J., Brinster, R.L., and Palmiter, R.D. (1989). Genetically haploid spermatids are phenotypically diploid. Nature *337*, 373–376.

Chiarini-Garcia, H., Hornick, J.R., Griswold, M.D., and Russell, L.D. (2001). Distribution of type A spermatogonia in the mouse is not random. Biol. Reprod. *65*, 1179–1185.

de Rooij, D.G., and Russell, L.D. (2000). All you wanted to know about spermatogonia but were afraid to ask. J. Androl. *21*, 776–798.

Erickson, B.H. (1981). Survival and renewal of murine stem spermatogonia following 60Co gamma radiation. Radiat. Res. *86*, 34–51.

Fuller, M.T., and Spradling, A.C. (2007). Male and female Drosophila germline stem cells: two versions of immortality. Science *316*, 402–404.

Hermann, B.P., Sukhwani, M., Hansel, M.C., and Orwig, K.E. (2010). Spermatogonial stem cells in higher primates: are there differences from those in rodents? Reproduction *139*, 479–493.

Hofmann, M.C., Braydich-Stolle, L., and Dym, M. (2005). Isolation of male germ-line stem cells; influence of GDNF. Dev. Biol. *279*, 114–124.

Huckins, C. (1971). The spermatogonial stem cell population in adult rats. I. Their morphology, proliferation and maturation. Anat. Rec. *169*, 533–557.

Huckins, C., and Oakberg, E.F. (1978). Morphological and quantitative analysis of spermatogonia in mouse testes using whole mounted seminiferous tubules, I. The normal testes. Anat. Rec. *192*, 519–528.

Kawamoto, S., Niwa, H., Tashiro, F., Sano, S., Kondoh, G., Takeda, J., Tabayashi, K., and Miyazaki, J. (2000). A novel reporter mouse strain that expresses enhanced green fluorescent protein upon Cre-mediated recombination. FEBS Lett. *470*, 263–268.

Klein, A.M., and Simons, B.D. (2011). Universal patterns of stem cell fate in cycling adult tissues. Development *138*, 3103–3111.

Klein, A.M., Nakagawa, T., Ichikawa, R., Yoshida, S., and Simons, B.D. (2010). Mouse germ line stem cells undergo rapid and stochastic turnover. Cell Stem Cell *7*, 214–224.

Lin, H., and Spradling, A.C. (1997). A novel group of pumilio mutations affects the asymmetric division of germline stem cells in the Drosophila ovary. Development *124*, 2463–2476.

Meistrich, M.L., and Van Beek, M.E. (1993). Cell and Molecular Biology of the Testis, C. Desjardins and L.L. Ewing, eds. (New York: Oxford Univ. Press), pp. 266–295.

Morrison, S.J., and Spradling, A.C. (2008). Stem cells and niches: mechanisms that promote stem cell maintenance throughout life. Cell *132*, 598–611.

Nakagawa, T., Nabeshima, Y., and Yoshida, S. (2007). Functional identification of the actual and potential stem cell compartments in mouse spermatogenesis. Dev. Cell *12*, 195–206.

Nakagawa, T., Sharma, M., Nabeshima, Y., Braun, R.E., and Yoshida, S. (2010). Functional hierarchy and reversibility within the murine spermatogenic stem cell compartment. Science *328*, 62–67.

Oakberg, E.F. (1971). Spermatogonial stem-cell renewal in the mouse. Anat. Rec. *169*, 515–531.

Ohbo, K., Yoshida, S., Ohmura, M., Ohneda, O., Ogawa, T., Tsuchiya, H., Kuwana, T., Kehler, J., Abe, K., Schöler, H.R., and Suda, T. (2003). Identification and characterization of stem cells in prepubertal spermatogenesis in mice. Dev. Biol. *258*, 209–225.

Rompolas, P., Mesa, K.R., and Greco, V. (2013). Spatial organization within a niche as a determinant of stem-cell fate. Nature *502*, 513–518.

Russell, L., Ettlin, R., Sinha Hikim, A., and Clegg, E. (1990). Histological and Histopathological Evaluation of the Testis. (Clearwater, FL: Cache River Press).

Sada, A., Suzuki, A., Suzuki, H., and Saga, Y. (2009). The RNA-binding protein NANOS2 is required to maintain murine spermatogonial stem cells. Science *325*, 1394–1398.

Sheng, X.R., and Matunis, E. (2011). Live imaging of the Drosophila spermatogonial stem cell niche reveals novel mechanisms regulating germline stem cell output. Development *138*, 3367–3376.

Shinohara, T., Orwig, K.E., Avarbock, M.R., and Brinster, R.L. (2000). Spermatogonial stem cell enrichment by multiparameter selection of mouse testis cells. Proc. Natl. Acad. Sci. USA *97*, 8346–8351.

Spradling, A., and Fan, C.M. (2010). Counterfeiting the family jewels. Cell Stem Cell *6*, 405–406.

Stine, R.R., and Matunis, E.L. (2013). Stem cell competition: finding balance in the niche. Trends Cell Biol. *23*, 357–364.

Suzuki, H., Sada, A., Yoshida, S., and Saga, Y. (2009a). The heterogeneity of spermatogonia is revealed by their topology and expression of marker proteins including the germ cell-specific proteins Nanos2 and Nanos3. Dev. Biol. *336*, 222–231.

Suzuki, M., Moriguchi, T., Ohneda, K., and Yamamoto, M. (2009b). Differential contribution of the Gata1 gene hematopoietic enhancer to erythroid differentiation. Mol. Cell. Biol. *29*, 1163–1175.

Uesaka, T., Jain, S., Yonemura, S., Uchiyama, Y., Milbrandt, J., and Enomoto, H. (2007). Conditional ablation of GFRalpha1 in postmigratory enteric neurons triggers unconventional neuronal death in the colon and causes a Hirschsprung's disease phenotype. Development *134*, 2171–2181.

Yoshida, S. (2012). Elucidating the identity and behavior of spermatogenic stem cells in the mouse testis. Reproduction *144*, 293–302.

Yoshida, S., Takakura, A., Ohbo, K., Abe, K., Wakabayashi, J., Yamamoto, M., Suda, T., and Nabeshima, Y. (2004). Neurogenin3 delineates the earliest stages of spermatogenesis in the mouse testis. Dev. Biol. *269*, 447–458.

Yoshida, S., Sukeno, M., and Nabeshima, Y. (2007). A vasculature-associated niche for undifferentiated spermatogonia in the mouse testis. Science *317*, 1722–1726.

Zheng, K., Wu, X., Kaestner, K.H., and Wang, P.J. (2009). The pluripotency factor LIN28 marks undifferentiated spermatogonia in mouse. BMC Dev. Biol. *9*, 38.

Cell Stem Cell, Volume *14*

Supplemental Information

Mouse Spermatogenic Stem Cells Continually Interconvert between Equipotent Singly Isolated and Syncytial States

Kenshiro Hara, Toshinori Nakagawa, Hideki Enomoto, Mikiko Suzuki, Masayuki Yamamoto, Benjamin D. Simons, and Shosei Yoshida

1. Supplemental Figures and Legends

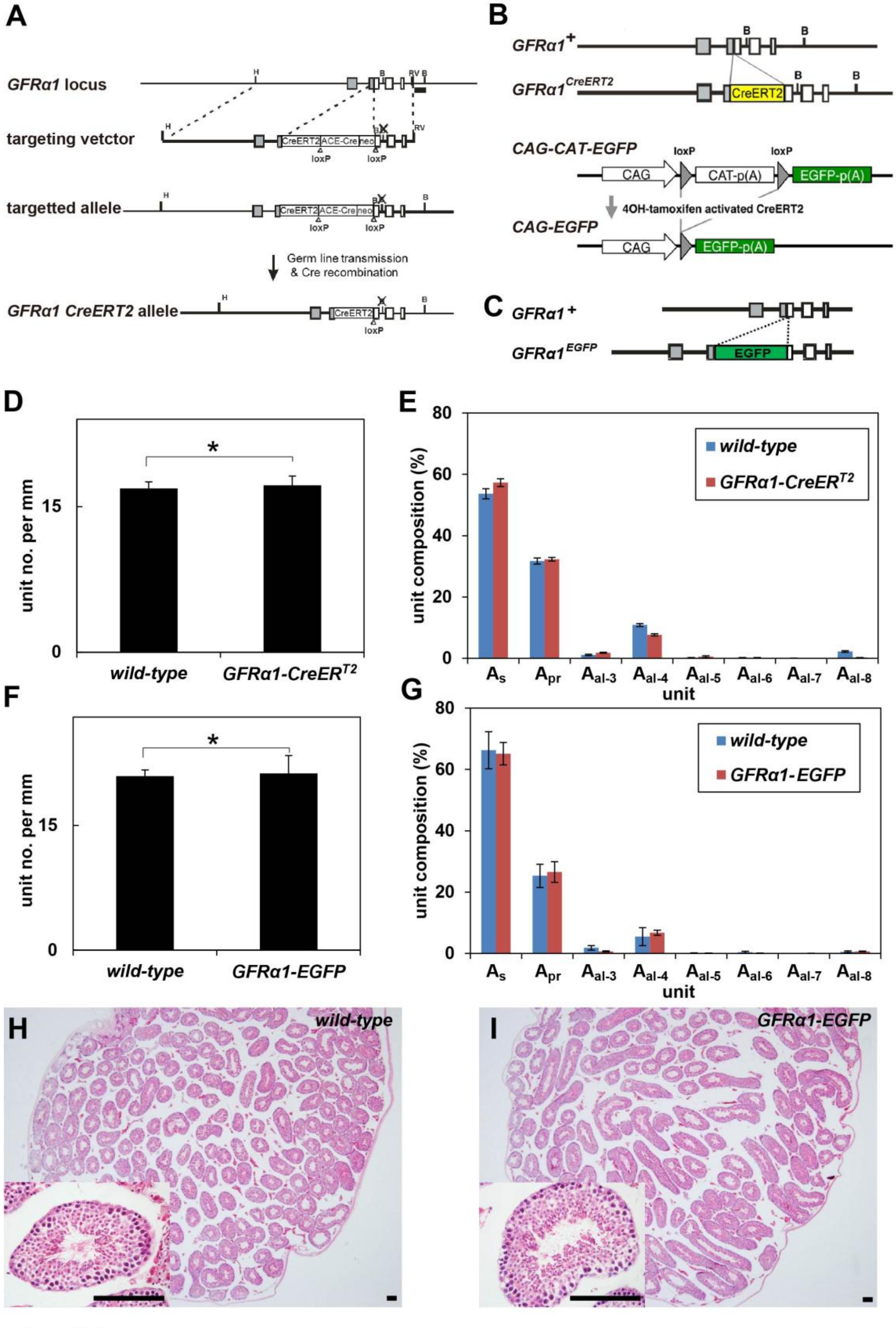

Fig. S1

Fig. S1: Supplemental data for the animals used in this study, related to Figs. 2, 3, 5, 6, and Experimental Procedures.

(A) Generation of *GFRα1-CreER*^{T2} Knock-in allele. A gene cassette composed of *CreER*^{T2} *cDNA-BGH poly(A)* followed by a floxed *angiotensin converting enzyme (ACE)-Cre* and *Tn5-neo* cassette were introduced into the second exon of the *GFRα1* gene by gene targeting as described previously (Enomoto et al., 2004). This insertion deleted 95 nucleotides containing the 5'-UTR, the initiator Met and the signal sequences. Upon germ line transmission of the targeted allele, floxed *ACE-Cre* and *Tn5-neo* were self-excised by ACE-Cre-mediated recombination in ES-cell-derived sperm (Bunting et al., 1999), culminating in the generation of *GFRα1-CreER*^{T2} allele. All animal procedures regarding the generation and characterization of *GFRα1-CreER*^{T2} allele were conducted with approval of the Washington University Animal Studies Committee and Animal Research Committee of the RIKEN Center for Developmental Biology. **(B)** Structure of *GFRα1* alleles and *CAG-CAT-EGFP* transgene. Injection of 4OH-tamoxifen into *GFRα1-CreER*^{T2}*; CAG-CAT-EGFP* mice induces temporal Cre activity in GFRα1+ cells, which causes permanent expression of *GFP* under *CAG* promoter, following excision of *CAT* gene floxed by the loxP sequences. **(C)** Structure of the *GFRα1-EGFP* knock-in allele carried heterozygously by the mice used for live-imaging (Uesaka et al., 2007). **(D-G)** No obvious effect of heterozygousity in *GFRα1* locus on the average GFRα1+ unit number (D, F) and composition (E, G) in 3-month-old *GFRα1-CreER*^{T2} (D, E) and 12-month-old *GFRα1-EGFP* (F, G) mouse testes. Average ± SEM (n=3), *p>0.05, Student's t test. **(H, I)** Hematoxylin/Eosin stained section of 12-month-old *wild-type* (H) and *GFRα1-EGFP* (I) testis, suggesting that *GFRα1* heterozygosity does not affect the overall integrity of spermatogenesis. Inset: higher magnified image of seminiferous tubule. Bars are 100μm.

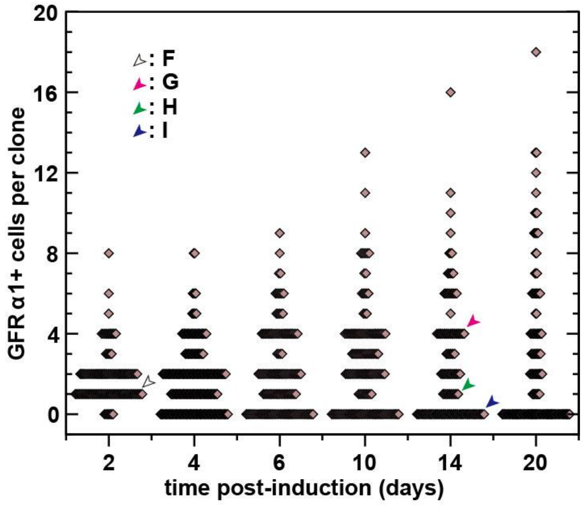

Fig. S2

Fig. S2: Supplemental data for fate analyses of pulse-labeled GFRα1+ spermatogonia clones in steady-state, related to Fig.2

Evolution of pulse-labeled clone size indexed by the number of GFRα1+ cells over 20 days. Data were obtained from 3, 4, 5, 4, 4, 6 and 3 testes at 2, 4, 6, 10, 14, and 20 days post-induction, respectively, summarized from the original data shown in Table S1. The clones shown in Fig. 2F-I are plotted as shown by white, magenta, green, and blue arrowheads, respectively.

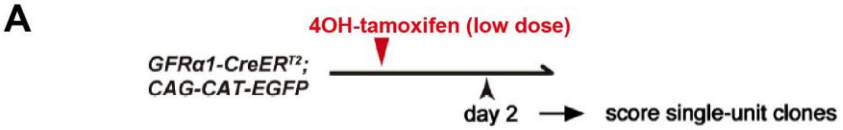

	total units	GFRα1+ units	GFRα1- units (observed)	GFRα1- units * (expected)	P value **
As	88	85	3	5.40	0.1280
Apr	53	48	5	3.25	0.2379
Aal-3	1	1	0	0.06	0.8038
Aal-4	19	18	1	1.17	0.8703
Aal-8	2	1	1	0.12	0.0117
total	163	153	10	10	

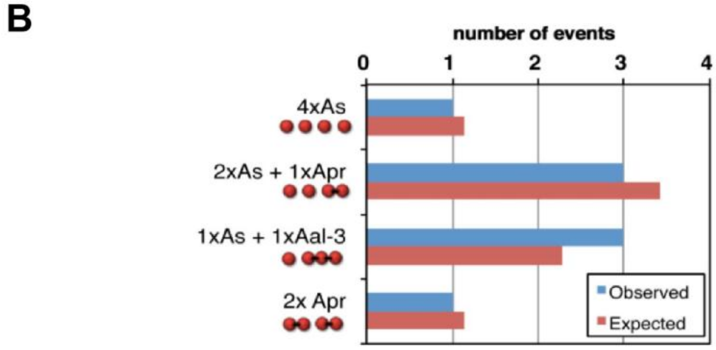

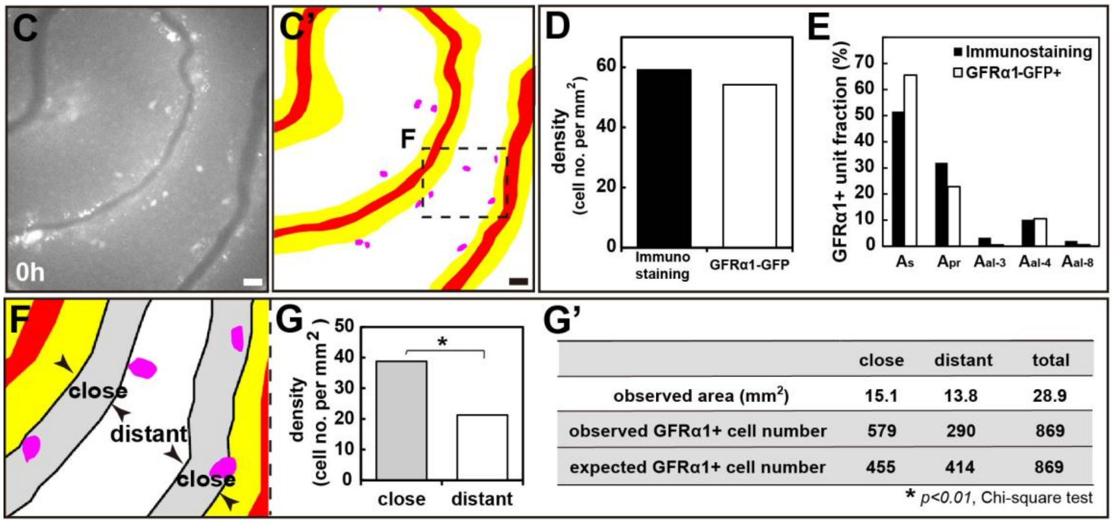

G'

	close	distant	total
observed area (mm^2)	15.1	13.8	28.9
observed GFRα1+ cell number	579	290	869
expected GFRα1+ cell number	455	414	869

* *p<0.01*, Chi-square test

Fig. S3

Fig. S3: Supplemental data for live-imaging analyses of GFRα1+ spermatogonia, related to Fig.3

(A) Transition of GFRα1+ A_s, A_{pr} and A_{al} spermatogonia into GFRα1– cells, analyzed by short-term pulse-labeling fate analysis. As shown on the top, GFRα1+ spermatogonia were sparsely labeled in 3-month-old mice. 2 days later, induced clones were analyzed for GFRα1 expression, and those comprising a single spermatogonial unit were scored. The composition of pulse-labeled GFRα1– units roughly reflects that of spermatogonia that underwent GFRα1+ → Ngn3+ transition, while this could be biased to longer units to some extent because some of them may have passed a cell division that doubled the syncytial length after transition to Ngn3+ in this short period. Therefore, this result indicates that differentiation occurs in all the entities of spermatogonial units. *Expected numbers of GFRα1– units are calculated by dividing the total number of GFRα1– units (viz. ten) according to the proportion of the total GFRα1+ units, assuming that all the GFRα1+ units had the same probability to transit into GFRα1–, and that GFRα1– units did not divide after losing GFRα1 expression. **P-values were obtained by Chi-square test between observed and expected numbers of GFRα1– units for each entity compared with all of the other units. Counts from 6 testis samples were summarized. **(B)** Fragmentation patterns of GFRα1+ A_{al-4} syncytia: In live-imaging study, all the possible patterns of fragmentation were observed at the indicated frequencies as shown in Fig. 3A (blue), which show a nice agreement with predicted values (red) (p=0.958 in Chi-square test). This prediction is done based on the assumption that each of the three intercellular bridges independently has 50% probability to break in an A_{al-4} syncytium that is licensed to fragment. In this case, out of seven possible permutations in which at least one intercellular bridge break, 1, 3, 2, and 1 cases give rise to $4xA_s$, $2xA_s$ + $1xA_{pr}$, $1xA_s$ + 1x A_{al-3}, $2xA_{pr}$, respectively. The expected frequencies are provided by allocation of the total 8 events according to these probabilities. **(C-E)** Density and composition of GFRα1-EGFP+ units observed by live-imaging in the surface area of mouse testes, compared with that of the tissue averages based on the whole-mount immunostained untangled seminiferous tubules. (C, C') Representative image obtained from the testis surface of *GFRα1-EGFP* mice (the first frame of Movie S4). (C') indicates the position of GFRα1+ spermatogonia (magenta), blood vessels (red) and interstitium (yellow) (C and C' are from the same image used in Fig.3E). (D, E) GFRα1+ cell densities (D) and unit fractions (E) counted by whole-mount immunostaining (black bars, data are reproduced from Fig.1D) and GFRα1-GFP fluorescence in seminiferous tubules located in surface area of *GFRα1-GFP* mouse testes (white bars). Counts from 3 testis samples were summarized. **(F-G')** Preferential localization of GFRα1+ spermatogonia near vasculature. (F) High magnification of the rectangular area in (C'), showing seminiferous tubule area divided into regions close to (<40μm; gray) and distant (>40μm; white) from the border of interstitium. (G) Density of GFRα1+ spermatogonia in regions close to and distant from the vasculature. Total density of GFRα1+ cells observed in 3 testes is shown. (G') Count of GFRα1-GFP+ cells observed in areas close to and distant from interstitium were significantly different from the expected distributions on the assumption of non-

biased distribution (*p<0.01, Chi-square test). Counts from 3 testis samples were summarized.

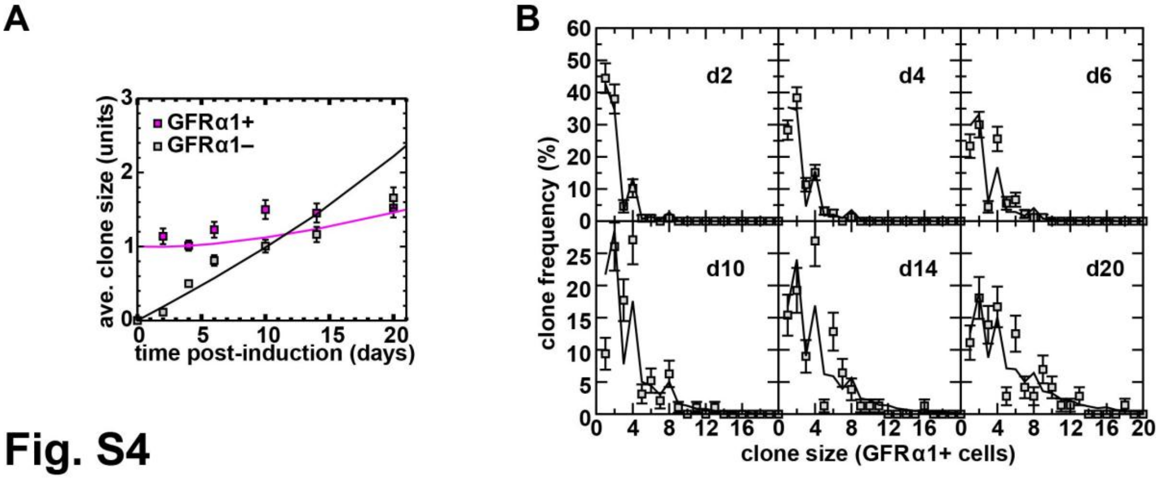

Fig. S4

Fig. S4: Supplemental data for model prediction of clonal fate behavior of GFRα1+ cells in steady state, related to Fig.4

(A) Capture of the average number of GFRα1+ (magenta) and GFRα1– (gray) units from the total clones observed *in vivo* (squares, average ± SEM among testes) by the model prediction (solid lines). **(B)** Capture of the distribution of GFRα1+ cell number in individual pulse-labeled clones scored *in vivo* (squares, average ± SEM among testes) by that predicted *in silico* (solid lines). *In vivo* data are reproduced from Fig.2L and Fig.S2, respectively.

A

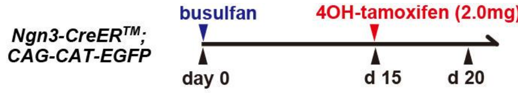

% labeling efficiency of Ngn3+ cells (a)	% contribution of labeled cells in GFRα1+ cells (b)	% contribution of Ngn3+ cells in GFRα1+ cells (c)	No. of GFRα1+ cells per mm tubules (d)	% contribution of Ngn3+ cells to steady-state value of GFRα1+ spermatogonia (e)
17.4 ± 6.5 ***	1.8 ± 0.3 ***	15.6 ± 5.3 ***	11.3 ± 0.9 ***	11.2 ± 4.7 ***

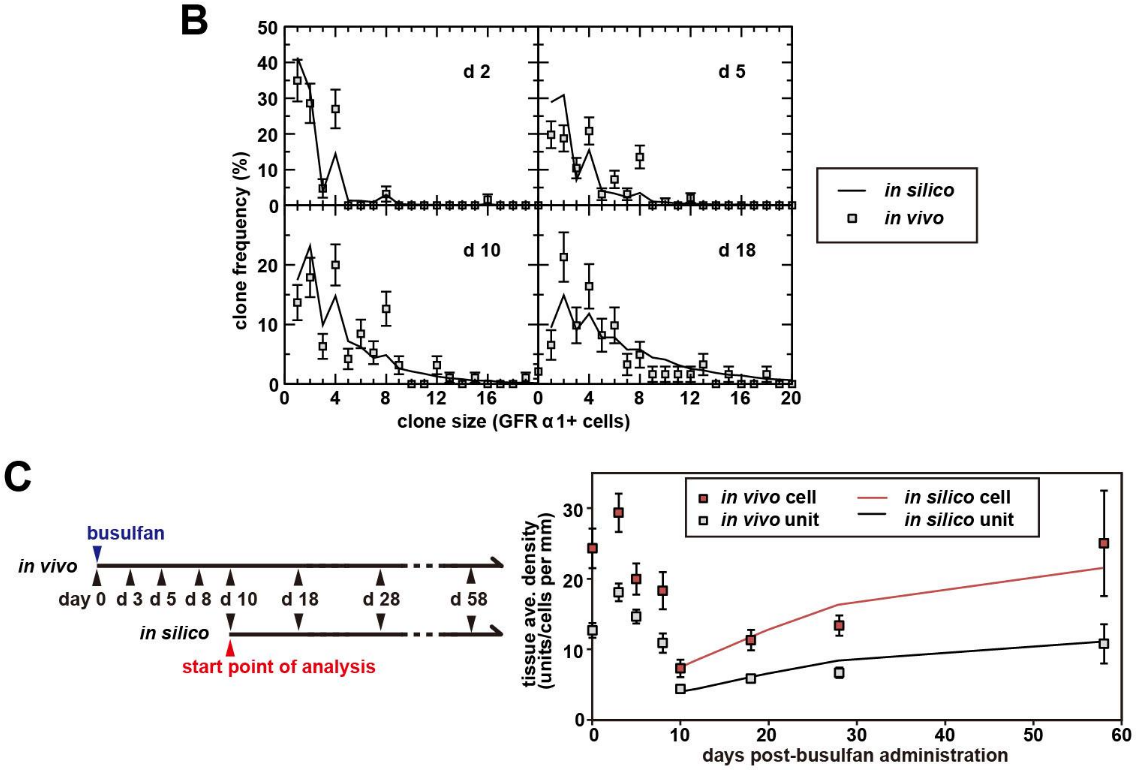

Fig.S5

Fig. S5: Supplemental data for the fate behavior of GFRα1+ spermatogonia in regeneration, related to Fig.6

(A) Contribution of Ngn3+ spermatogonia into the GFRα1+ compartment during the period of regeneration. As shown on the top, by using *Ngn3-CreER^{TM}; CAG-CAT-EGFP* mice, Ngn3+ cells were labeled by 4OH-tamoxifen administration during the regeneration process 15 days after busulfan injection, followed by the analyses of labeled cells 5 days later. (a) Labeling efficiency of Ngn3+ cells was estimated as the contribution of labeled cells to Kit+ differentiating spermatogonia observed in stages IX-XI on day 20, which had differentiated from undifferentiated spermatogonia that were Ngn3+ at the time of 4OH-tamoxifen administration. (b) Contribution of labeled cells to the total GFRα1+ spermatogonia on day 20. (c) Estimated contribution of cells that were Ngn3+ on day 15 to the GFRα1+ population on day 20 was calculated as (b)/(a). (d) Number of total GFRα1+ cells per mm tubules. (e) Contribution of Ngn3+ cells to the steady-state density of GFRα1+ cells (17 units/mm) during the 5 days, calculated

(c)*(d)/17. Numbers indicate average ± SEM from 4 testes. **(B)** Clone size distribution indexed by the number of GFRα1+ cells in individual clones in regeneration by the model using optimized parameters. As in Fig. 6, experimental data (squares) are shown as average ± SEM. *In silico* predictions are shown by solid lines. **(C)** Evolution of tissue average density of GFRα1+ spermatogonia during regeneration over two months. Left panel shows the time-schedule of *in vivo* data acquisition after busulfan administration (designated as day 0) and *in silico* analysis. Right panel shows the measurements of the tissue average density of GFRα1+ units/cells observed *in vivo* (squares) and the *in silico* model prediction (lines). Experimental data are reproduced form Nakagawa et al., 2010 and shown as average ± SEM from 3 testes at each time-point.

2. Supplemental Experimental Procedures

2-1. Intravital live imaging and image analysis

Live-imaging of the testes of four- to five-month-old *GFRα1^{EGFP}* or *GFRα1^{EGFP};GATA1-EGFP* mice under anesthesia was performed for three days as described before, using epifluorescence IX61WI microscope (Olympus) (Yoshida et al., 2007). Time-lapse images were captured at the rate of one flame per 30 minutes using the Andor iXon EM-CCD camera controlled by Metamorph software (Molecular Devices). Movies were constructed by Metamorph software, and the trajectories of spermatogonia and Sertoli cells were manually extracted from the movies using the Metamorph and ImageJ softwares.

To classify complete and incomplete cell divisions, an intercellular bridge was deemed to be intact if the cells remained within 30μm for more than 12 hours, on the basis of the following observations. In the live-imaging study, we observed 35 divisions of A_s cells in total. In 4 cases, the resultant daughter cells subsequently separated into two A_s cells by the end of the record, whereas the daughter cells remained associated until the end of the record in the other 31 cases. The intervals between the division of parental A_s cell and the separation of the daughters in these 4 cases were 4, 11, 37, and 43 hours. Accordingly, we practically defined an A_s division as a "complete division", when the separation of the daughter cells were observed within 12 hours following division. So, the two cases with 4 and 11 hours of intervals were classified as complete divisions, while we categorized the other two cases (with 37 and 43 hours of intervals) as sequences of incomplete division followed by syncytial fragmentation. We set such a strict criterion so that we could avoid miscategorizing a complete division as "false incomplete division", while "false complete division" could be accepted to some extent (such as the one with 11 hours of interval after division).

2-2. Biophysical model

To explore and interpret the clonal dynamics of the GFRα1+ population, we developed a minimal biophysical modeling scheme, as described concisely in the main text. In this supplemental section, we set out in more detail the basis of the model and its practical implementation.

1) Framework of the modeling scheme

In accordance with the literature (Nakagawa et al., 2010; Sada et al., 2009; Yoshida, 2012), the result of the pulse-labeling experiment in the current study suggests that the population of GFRα1+ spermatogonia is primarily responsible for the stem cell activity (Fig. 2). In addition, the current live-imaging study revealed that GFRα1+ spermatogonia continually and reversibly interconvert between different state of A_s, A_{pr} and various lengths of A_{al} spermatogonia (Fig. 3). Therefore, we developed a biophysical modeling

scheme based on the assumption that stem cell activity was restricted to, and defined by the entirety of, the pool of morphologically heterogeneous GFRα1+ spermatogonia. In the model, for simplicity, the GFRα1+ → Ngn3+ transition was considered irreversible resulting in the permanent loss of GFRα1+ cells towards differentiation. Indeed, although the Ngn3+→GFRα1+ reversion has been observed, the preceding (as well as the current) studies showed that the frequency of this process is very low in steady state (Nakagawa et al., 2010, and Fig. 2D). In any case, taking into account the small fraction of Ngn3+ cells that transit back into the GFRα1+ compartment would not significantly affect the quantitative predictions of the model.

The GFRα1+ spermatogonial units are shown to be dispersed and actively moving over the tubules. Intriguingly, however, their local density, averaged over several millimeters of tubule, was found to be reproducibly constant. To reflect this situation, we proposed a regular quasi one-dimensional lattice model, in which each domain accommodates a single GFRα1+ unit, providing a caricature of the basal compartment of the seminiferous tubule (Fig. 4A). The lattice spacing along the longitudinal axis and around the circumference (1/3 mm in length and 1/5 of circumference) was chosen to be commensurate with the measured average density and their preferential localization to the vasculature (see main text). In this scheme, the territories of individual GFRα1+ units, which *in vivo* should be variable and even dynamic in their shape, area and arrangement, are considered even and regular in the tubules. Although this assumption of a regular lattice geometry is an obvious over-simplification, features sensitive to this approximation are expected to be small and beyond the resolution of our analysis.

Alongside the generation of Ngn3+ cells, the live-imaging study (Fig. 3A) showed that GFRα1+ units may undergo two further distinct processes: they may extend in unit length through incomplete cell division (Fig. 4B), and they may undergo multiplication through syncytial fragmentation (Fig. 4C). Based on the results of the live-imaging study (Fig. 3A), we supposed that the contribution made by the process of complete A_S cell division is sufficiently small that it may be neglected. Similarly, for simplicity, we neglected the apparently small contribution made by cell death of GFRα1+ cells.

In this scheme, to maintain the stem cell pool size (viz. the density of GFRα1+ units), the syncytial fragmentation (which multiplies GFRα1+ units) was correlated with the loss of a GFRα1+ unit (through transition to a Ngn3+ unit) on a neighboring domain. Practically, we supposed that, following fragmentation, one of the fragments stays at the "mother" site, while the others migrate to any one of the neighboring sites, where the existing GFRα1+ unit is lost through becoming Ngn3+ at that site.

2) Defining the constituent cell behavior

Even within the scope of such a simplified modeling scheme, we can contemplate numerous potential sources of variability that would increase the complexity of the model, in relation to the aforementioned cellular processes (viz. cell division, syncytial

fragmentation, and transition into being Ngn3+). For example, within the GFRα1+ population, the rates of any of these processes may depend sensitively on syncytial length. Similarly, syncytial fragmentation may cause the binary fission of a syncytium due to the breakage of a single intercellular bridge, or it may fragment into three or more components following the breaking of multiple bridges. Moreover, the fragmentation frequency may depend on the relative position of the bridge within the syncytium itself (which is related to the seniority of the bridge since its generation by incomplete cell division). Furthermore, the migration of syncytia between sites may be spatially anisotropic and it may involve transfer of syncytia over multiple lattice spacings. In addition, the behavior of cells after becoming GFRα1– may be correlated with the differentiation steps (Ngn3+, Kit+, or more advanced cell types).

Fortunately, we could take advantage of the observations from the live-imaging study to constrain the variability of the aforementioned cellular dynamics, which subsequently reduces the multitude of parameters *in silico*. In doing so, we found that the complex clonal dynamics observed *in vivo* conforms to a remarkably simple paradigm of the modeling scheme, as seen below.

First, from the results of the live-imaging study, it was apparent that the average GFRα1+ cell division rate, *D*, of approximately once per 10 days, is largely independent of the unit length (viz. regardless of being A_s, A_{pr} or A_{al}) (Fig. 3A). Second, although the frequency of fragmentation of GFRα1+ syncytia appeared to increase with unit size, the observed dependence could be captured by assuming that the rate correlates linearly with the number of intercellular bridges, which enabled the setting of a single rate constant of syncytial fragmentation (*F*) at once per 20 days per intercellular bridge. Moreover, the live-imaging data also suggested that any one of the bridges may break with around a 50% probability, once a syncytium is licensed to fragment (Fig. 3A, Fig. S3B).

Although the live-imaging data constrains the average rates of cell division and fragmentation as described above, the distribution of timings between the same or different events is, as yet, unspecified. In the following, for simplicity (to minimize the number of parameters), we assumed a Markov process in which the timings between consecutive events (cell division and fragmentation) were stochastic and statistically uncorrelated. In particular, these processes were drawn at random from an exponential distribution (Poisson process), with the defined average rates. While such an assumption was convenient, features associated with any degree of correlation between these processes, which may be present *in vivo*, would be rapidly erased from the clonal records.

Recognizing the role of the vasculature in specifying a facultative niche environment (Yoshida et al., 2007), we further assumed that some 70% of unit replacements occur along the axis of the tubule, while only 30% involve transfer across the tubule. Although this bias acknowledges the role of the vasculature in guiding the movement of GFRα1+ spermatogonia along the tubule axis, the properties of the model do not depend sensitively on the precise ratio. Further, based on the observed motility

of spermatogonial units in live-imaging (Fig. 3E-H), we allowed approximately 1/7 of replacements along to the tubule direction to occur at next-nearest neighbor sites. Once again, the model dynamics depends only weakly on this parameter.

Finally, the literature (Huckins and Oakberg, 1978) and our unpublished live-imaging observations indicated that GFRα1– (Ngn3+ and Kit+) units die to some degree. It is clear, however, that the death of GFRα1– units is not a stochastic event but preferentially observed in particular cell types that appear during the differentiation process. Nevertheless, since this event does not affect the dynamics of the surviving GFRα1+ population per se, we supposed that the death of GFRα1– cells also follow a Poisson process with a defined rate. Practically, we chose a death rate of around once per 30 days, which was consistent with the observations.

On extrapolating this model to predict longer-term clonal behavior over months to over a year, which was based on the measured clone length along the tubule axis and the number of the surviving clonal patches, it was supposed the differentiating cohort of GFRα1+ spermatogonia fully occupy domains of the lattice (which corresponds to the territory of an individual GFRα1+ unit) after a delay of approximately two weeks, during which time they mitotically amplify and reach the stage of spermatocytes. To reflect this, *in silico*, the length and number of the patches were predicted based on the occupation of domains by GFRα1+ spermatogonia two weeks in advance.

3) Characterization of the model dynamics and prediction of the *in vivo* observation

Despite the involvement of multiple components in this scheme, the dynamics of clonal evolution is essentially specified by just two parameters: the cell division rate, D, and the syncytial fragmentation rate per intercellular bridge, F. Moreover, in steady-state, the unit composition depends only on the ratio of these parameters, D/F. To investigate the clonal dynamics predicted by the model, one could consider the development of an analytical approach based on the analysis of a Master equation describing the time-evolution of the clone probability distribution (see, e.g., Lopez-Garcia et al., 2010). However, as a quasi one-dimensional system involving multiple components, the resulting Master equation would be analytically intractable. Fortunately, however, the steady-state unit composition and clone size distribution, both of which were independent measurements from those used to build up the model, could be recovered accurately from a straightforward numerical stochastic simulation of the dynamics of GFRα1+ spermatogonia under the foregoing modeling scheme.

Operationally, to follow the clonal dynamics of the GFRα1+ cell population, we began by seeding the lattice with a random configuration of GFRα1+ units in proportions that reflect the measured unit composition. To clonally trace these units, each was given a unique “barcode” which was then inherited by their progeny. The system was then allowed to evolve according to the dynamics specified above. As GFRα1+ units were lost through transition into being Ngn3+ and replaced following syncytial fragmentation, some clones that survived expanded while others became extinct by losing all the

GFRα1+ units. When GFRα1+ units were lost and became GFRα1–, a record was kept of their unit number on each domain. We did not, however, attempt to follow the expansion of GFRα1– units through cell division.

According to the defined dynamics, the model will converge from any arbitrary initial composition of spermatogonial units to a particular steady-state composition, which depends uniquely on the ratio of the cell division to fragmentation rates, D/F (Fig. 4D). Using the D and F rates of once per 10 days and once per 20 days per bridge, respectively, obtained directly from the live-imaging study (Fig. 3A), the model could predict the composition of the observed steady-state average of the GFRα1+ units with remarkable accuracy (Figs. 4D, E). We then used the computational scheme to predict the steady-state clonal evolution over 20 days following pulse-labeling. Comparison of the *in silico* prediction with the wide range of *in vivo* clonal fate data indexed by the number of GFRα1+ and GFRα1– spermatogonia within a clone revealed a surprisingly good agreement for both unit and cell number, over the entire 20 day time course (Figs.4G,H,I and Figs.S4A,B). Considering that the steady-state composition of GFRα1+ units and the clonal fates of pulse-labeled GFRα1+ units are totally independent of the data that were used to build up the modeling scheme, these agreements strongly support the validity of the modeling scheme. In addition, the validity of the model was further supported by its ability to predict the long-term (for months to over a year) clonal fates, and the dynamics in regeneration, as described in depth in the main text (Figs. 5, 6 and S5BC).

It is interesting to note that, by correlating syncytial fragmentation with the loss of GFRα1+, in steady-state, the effective rate of GFRα1+→Ngn3+ transition must be equal, by definition, to the cell division rate (D). More precisely, over $1/D$ days (the average cell division period, corresponding to 10 days based on the current live-imaging study), the total cell number is precisely doubled. Given the steady-state dynamics, one half of these cells must exit the GFRα1+ compartment, while the other half remains GFRα1+ during this period. If the overall transition occurs in proportion to the steady-state composition of GFRα1+ units, the spermatogonia that have become GFRα1– over the $1/D$ days must be equal to that of the original GFRα1+ population, in both cell and unit numbers. Because transition to a GFRα1– unit is allied with replacement following a multiplication of the neighboring GFRα1+ unit, the above property also shows that the rate of replacement between neighboring domains should also be equal to the rate of cell division, or once per 10 days in particular. Of note, this agrees with the previous estimation of the replacement rate to be once per less than two weeks based on the long-term (months to over a year) fate analyses of Ngn3+ spermatogonia-derived patches (Klein et al., 2010).

The current model also recovers the scaling function, a hallmark behavior of the population asymmetry, which was observed in the long-term distribution of the clone size indexed by patch length (Fig. 5D)(Klein et al., 2010). These findings not only support the premise that the observed long-term behavior reflected that of GFRα1+ spermatogonia to which the labeled Ngn3+ cells were expected to revert, but also provide a cell-level explanation of the ongoing stochastic stem cell loss and replacement.

4) Consideration of alternative scenarios

Although the results in this study fully support the theory of single stem cell pool composed of functionally equivalent GFRα1+ A_s and syncytia, as implied by the proposed model (Fig. 7A), one may also conceive of alternative scenarios. For example, a small compartment of slow-cycling GFRα1+ A_s cells that act as the "true" stem cells might undergo rare asymmetrical division (an infrequent event corresponding to the low number of complete divisions that were observed). Then, one of the daughter A_s cells could replenish and maintain the stock of slow-cycling stem (A_s) cells. The other daughter A_s GFRα1+ cell would then transfer to the active GFRα1+ compartment. This second compartment, which is much larger than the former in number, would repeat incomplete division and syncytial fragmentation as observed in this study. However, cells in the second, active, compartment would have limited short-term longevity and, eventually, would become replaced by the A_s daughter from the slow cycling compartment to maintain long-term homeostasis. As described below, although we don't –and can't– rigorously rule out the presence of such a slow-cycling compartment, we can conclude that their contribution (would they exist) would not be essential for the maintenance of life-long spermatogenesis in mouse.

Indeed, the "equipotent model" (that proposed in this study) is by itself able to provide a highly accurate quantitative prediction of the short-term clonal fate data over the 20 day time course. However, it would be difficult to rule out alternative models such that described above solely on the basis of these short-term data. However, even if the "slow-cycling" model could capture aspects of the "short-term" dynamics (which is far from clear), the "long-term" (which we defined here as a time scale from months to over a year, which effectively covers the reproduction period of the mouse) behavior of the pulse-labeled GFRα1+ spermatogonia provides a key for discrimination between these models.

Importantly, the "equipotent" model, which is synthesized solely from measurements in the live-imaging study (up to 3 days), quantitatively predicts not only the short-term (up to 20 days) but also the long-term (up to 14 months) clonal behavior (viz. continuous clonal loss and the size distribution of surviving clones: Fig. 5). Of particular note, the predicted long-term behavior does not require any adjustment of the model, but is achieved by its simple extrapolation to long times. In other words, both short- and long-term behaviors are fully explained by this same minimal model, and do not require other factors (such as a "slow-cycling" A_s population) to explain the wide range of experimental observations. In this context, it is important to understand that the equipotent model faithfully recovers the "scaling" behavior of the clone size distribution over different time scales, a robust and parameter-independent hallmark of stochastic stem cell loss and replacement (Klein and Simons, 2011).

On the other hand, if one assumes that tissue is maintained long-term by a "slow-cycling" A_s cell compartment, then we would see two characteristic behaviors at different time scales. In the short term (relative to the turnover time of the slow cycling

cells), we would see the clonal depletion and the expansion of the surviving clones, reflecting the feature of short-lived “active” GFRα1+ cells that are positioned inferior to the long-lived “slow-cycling” GFRα1+ cells and destined to disappear from the tissue. After this transient phase of loss of clones derived from short-lived cells, we would then expect a transfer of the clonal dynamics to a new distinct phase, where rates of clonal loss and replacement should significantly decrease reflecting the persistence of clones originating from the “slow-cycling” A_s cells. Contrary to this expectation, in reality, the observed fate behavior of GFRα1+ cells follows from a single dynamics (again, defined by just two rates measured from the very-short-term live-imaging study), in both short- and long-term. Therefore, the contribution of a slow-cycling stem cell population, which is presumably located on the top of the hierarchy, is not quantitatively supported by the data. In addition, on the basis of the same experimental and mathematical evidence described above, if the population of GFRα1+ spermatogonia involve multiple subsets characterized by different kinetics, significant contribution of any kind of slowly-turning-over population is not supported.

3. Supplemental Table S1

The entire clonal fate raw data of pulse-labeled GFRα1+ spermatogonia in steady state, related to Fig. 2 (provided as a separate Excel file). Data are summarized from 3, 4, 5, 4, 4, 6 and 3 testes for steady-state measure and at 2, 4, 6, 10, 14, and 20 days post-induction, respectively.

4. Supplemental Movies

Movie S1: An example of $A_s \rightarrow 2x\ A_s$ division, supporting Fig. 3B
Movie S2: An example of $A_s \rightarrow A_{pr}$ division, supporting Fig. 3C
Movie S3: An example of cell division of $A_{pr} \rightarrow A_{al\text{-}4}$ followed by a fragmentation into an A_s and an $A_{al\text{-}3}$, supporting Fig. 3D
Movie S4: Prominent migration of GFRα1-EGFP+ spermatogonia, supporting Fig. 3E-F
Movie S5: Migration of GFRα1-EGFP+ spermatogonia between Sertoli cells revealed by in vivo live imaging of GFRα1-EGFP; GATA1-EGFP mouse testis, supporting Fig. 3G-H
Throughout, the time scale is shown as elapsed time in days: hours: minutes.

5. Supplemental References

Bunting, M., Bernstein, K.E., Greer, J.M., Capecchi, M.R., and Thomas, K.R. (1999). Targeting genes for self-excision in the germ line. Genes Dev *13*, 1524-1528.

Enomoto, H., Hughes, I., Golden, J., Baloh, R.H., Yonemura, S., Heuckeroth, R.O., Johnson, E.M., and Milbrandt, J. (2004). GFRalpha1 expression in cells lacking RET is dispensable for organogenesis and nerve regeneration. Neuron *44*, 623-636.

Lopez-Garcia, C., Klein, A.M., Simons, B.D., and Winton, D.J. (2010). Intestinal stem cell replacement follows a pattern of neutral drift. Science *330*, 822-825.
